# GPA33-pretargeted radioimmunotherapy with mono- and bivalent DOTA-based Lu-177-labeled radiohaptens in a mouse orthotopic liver xenograft model of metastatic human colorectal cancer

**DOI:** 10.7150/thno.107209

**Published:** 2025-05-25

**Authors:** Alexandre B. Le Roux, Edward K. Fung, Sang Gyu Lee, Sebastien Monette, Hong Xu, Hong-Fen Guo, Guangbin Yang, Ouathek Ouerfelli, Achim Jungbluth, Heiko Schöder, Steven M. Larson, Nai-Kong V. Cheung, Sarah M. Cheal, Darren R. Veach

**Affiliations:** 1 Department of Radiology, Memorial Sloan Kettering Cancer Center, New York, NY; 2 Department of Radiology, Weill Cornell Medicine, New York, NY; 3 Laboratory of Comparative Pathology, Memorial Sloan Kettering Cancer Center, Weill Cornell Medicine, and Rockefeller University, New York, NY; 4 Department of Pediatrics, Memorial Sloan Kettering Cancer Center, New York, NY; 5 Organic Synthesis Core Facility, Memorial Sloan Kettering Cancer Center, New York, NY; 6 Department of Pathology, Memorial Sloan Kettering Cancer Center, New York, NY; 7 Molecular Pharmacology Program, Memorial Sloan Kettering Cancer Center, New York, NY

**Keywords:** multivalent radiohapten, Lu-177, theranostics, colorectal cancer hepatic metastasis, pretargeted radioimmunotherapy

## Abstract

**Rationale:** Pretargeted radioimmunotherapy (PRIT), which combines systemic antibody-based targeting with ionizing radiation, is promising for treating liver metastases in patients with colorectal cancer (CRC). Previously, we established a three-step DOTA-PRIT regimen to deliver DOTA radiometal payloads to CRC using an anti-tumor/anti-DOTA bispecific antibody (BsAb) targeting cell surface glycoprotein A33 (GPA33), a tumor antigen target expressed on over 95% of primary and metastatic CRC; a clearing agent; and a monovalent ^177^Lu radiohapten called [^177^Lu]Lu-ABD. More recently, we developed a bivalent ^177^Lu radiohapten called [^177^Lu]Lu-Gemini to enhance tumor uptake and radiohapten retention. Here, we aimed to compare the efficacy and safety of bivalent vs. monovalent three-step DOTA-PRIT regimens in orthotopic CRC liver metastasis models, to mimic a clinical path forward.

**Methods:** We established two orthotopic CRC liver metastasis models by inoculating either SW1222-luc (GPA33^high^) or LoVo (GPA33^low^) human CRC cells in athymic nude mice under ultrasonographic guidance. Tumor targeting efficacy and dosimetry of the radiohaptens were compared using *ex vivo* biodistribution studies, SPECT/CT, and quantitative autoradiography. We also performed a DOTA-PRIT experiment to compare the efficacy and safety profiles of bivalent (single-cycle [^177^Lu]Lu-Gemini, 48 h pretargeting interval) vs. monovalent (multicycle [^177^Lu]Lu-ABD, 24 h pretargeting interval) three-step DOTA-PRIT regimens, each designed to deliver comparable total radiation doses to tumors (around 50 Gy).

**Results:** Both radiohaptens demonstrated efficient SW1222-luc tumor targeting, with [^177^Lu]Lu-Gemini showing superior targeting and tumor activity retention compared with [^177^Lu]Lu-ABD. In LoVo tumors, [^177^Lu]Lu-Gemini showed superior targeting, while [^177^Lu]Lu-ABD showed negligible targeting. Dosimetry estimates revealed higher SW1222-luc tumor mean absorbed doses for [^177^Lu]Lu-Gemini (119.88 cGy/MBq, 48 h pretargeting interval) compared with [^177^Lu]Lu-ABD (32.88 cGy/MBq, 24 h pretargeting interval), with more favorable blood and kidney therapeutic indices (50 and 9 for [^177^Lu]Lu-Gemini, and 15 and 5 for [^177^Lu]Lu-ABD, respectively). In the DOTA-PRIT experiment, both monovalent (injected activity: 3 × 44.4 MBq, 133.2 MBq total) and bivalent (injected activity: 44.4 MBq total) radiohapten regimens increased the median survival of treated mice compared with controls: 71 days for [^177^Lu]Lu-ABD-treated mice, 81 days for [^177^Lu]Lu-Gemini-treated mice, and 18 days for controls, without a statistical difference between treatment groups. Treatments were well tolerated, without significant weight loss or hematologic changes. Radiation-induced injuries were not identified histologically in the kidneys or bone marrow of mice submitted for necropsy.

**Conclusions:** Our study demonstrates the exceptional benefit of a multivalent radiohapten strategy when treating an advanced model of CRC liver metastasis. Three-step GPA33 DOTA-PRIT with ^177^Lu-Gemini demonstrated that multivalency 1) improves PRIT therapeutic indices for blood and kidney and 2) has the potential to greatly reduce the administered activity without compromising the efficiency of the PRIT platform in clinically relevant models of target-rich and target-poor metastatic CRC.

## Introduction

Tumor metastasis is the leading cause of mortality in patients with colorectal cancer (CRC), with the liver being the most common metastatic site, accounting for 70% of all cases 1-4. About 20-25% of patients with CRC already have metastasis at diagnosis, and an additional 20% will develop metachronous metastasis within three years after diagnosis, limiting surgical cure 5-7. Indeed, conventional therapies have shown limited efficacy for treating metastatic CRC 5, 7, 8. Thus, innovative systematic therapies are being developed as alternatives to conventional therapy to improve patient outcomes. In this context, pretargeted radioimmunotherapy (PRIT) is one such targeted treatment approach being developed that has shown promise in the experimental setting.

In PRIT, the slow antibody distribution phase is separated from the radioactive payload, thus enhancing tumor specificity while reducing off-target radiation exposure compared with conventional radioimmunotherapy using a radioimmunoconjugate 9. PRIT has shown success in targeting the A33 glycoprotein (GPA33), a tumor-associated transmembrane antigen expressed in 95% of primary and metastatic CRC 10-14. Our current anti-GPA33 β-DOTA-PRIT (“DOTA-PRIT”) regimen consists of three steps: the injection of an IgG-scFv anti-GPA33/anti-DOTA bispecific antibody (BsAb), followed 20 h later by the injection of a clearing agent to remove unbound BsAbs from the blood, and finally, 24 h after initial BsAb administration, by the injection of a monovalent radiohapten, [^177^Lu]Lu-ABD (also referred as [^177^Lu]Lu-DOTA-Bn in the literature). [^177^Lu]Lu-ABD is formed by a complex of lutetium-177 (^177^Lu)-labeled aminobenzyl-DOTA 12, 14-19. ^177^Lu is a β-emitter (maximum energy: 0.5MeV, average energy: 0.13 MeV) with a half-life of 6.65 days, and it also emits γ-radiation that can be imaged using single-photon emission tomography (SPECT). ^177^Lu has shown potential for radiotheranostics applications, especially for treating gastroenteropancreatic neuroendocrine tumors and metastatic castration-resistant prostate cancer 20.

**Figure 1:**
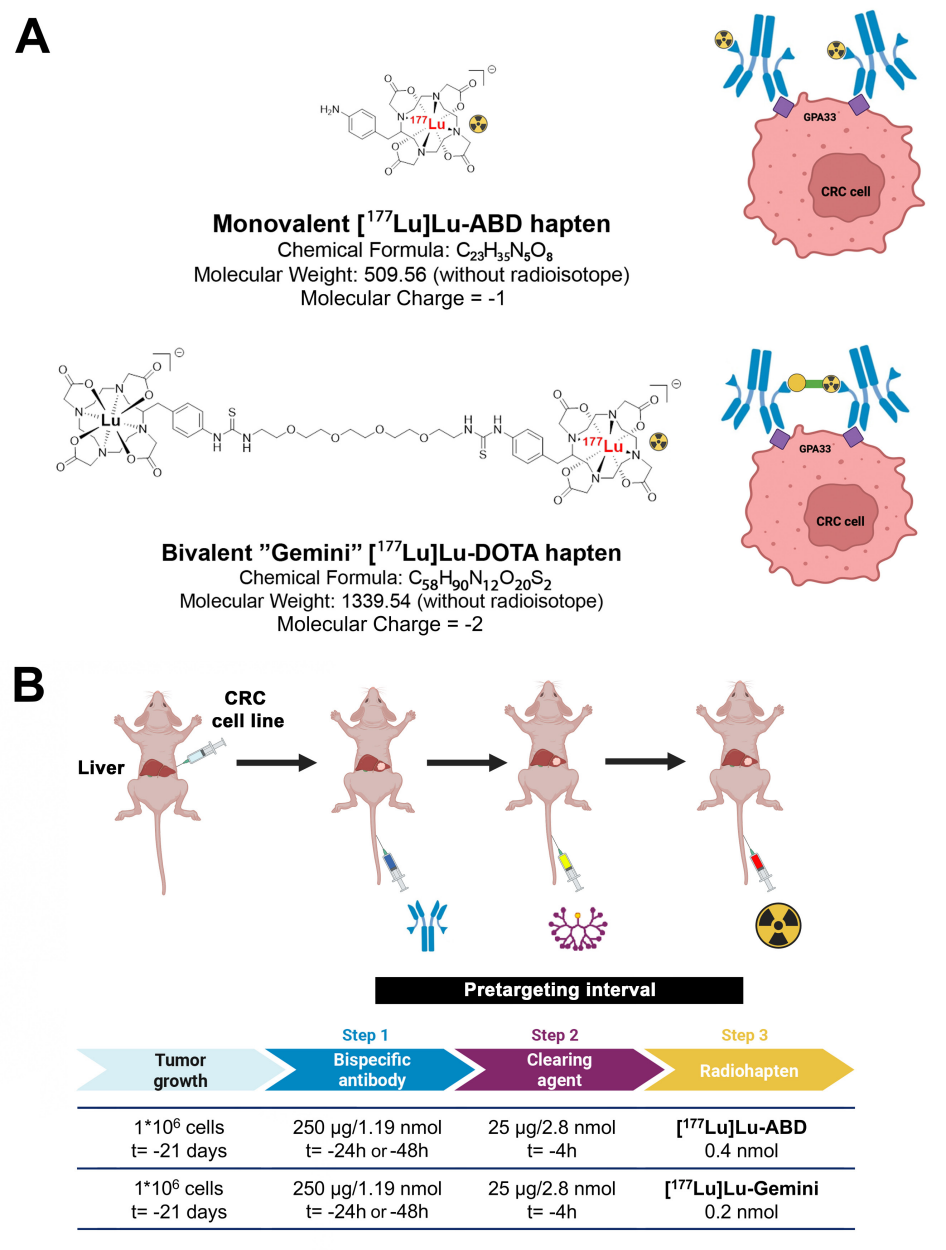
**DOTA-based pretargeted radioimmunotherapy (DOTA PRIT) using monovalent and bivalent ^177^Lu-labeled radiohaptens. A.** Concept of avidity enhancement. [^177^Lu]Lu-Gemini is a symmetrical dimer of ABD and forms highly stable complexes with a variety of radiometals (β and α-emitters). Gemini potentially allows cooperative binding, with both DOTA binding with the BsAb scFv C825 anti-(M) ABD site. The Gemini radiohapten precursor is initially radiometallated with ^177^Lu, and the remaining empty DOTA chelators then filled with nonradioactive ^175^Lu. Non-radioactive lutetium backfilling is necessary, as both DOTA must be metal bound (M-DOTA) to be recognized by the antibody on either side, allowing for cooperative binding and enhanced retention at the cell surface. **B.** Three-step DOTA PRIT protocol.

Notably, three-step DOTA-PRIT based on [^177^Lu]Lu-ABD has shown excellent therapeutic efficacy without treatment-related toxicities in subcutaneous GPA33-expressing human CRC xenograft mice 12-14, 19. However, looking toward clinical translation, while subcutaneous xenograft tumors are easy to grow, and while their progression and response to therapeutic intervention can be easily monitored non-invasively 21, 22, the subcutaneous tumor microenvironment does not fully mirror relevant clinical situations; correspondingly, subcutaneous xenograft models are considered suboptimal models, proven to be poor predictors of drug efficacy in humans 23. Hence, we have established an orthotopic CRC liver metastasis model using minimally invasive percutaneous ultrasound-guided intraparenchymal liver inoculation of two distinct CRC cell lines (GPA33^high^ SW1222-luc and GPA33^low^ LoVo cell lines), reflecting human CRC liver metastasis 24. Moreover, we have also more recently developed a bivalent ^177^Lu-based radiohapten, [^177^Lu]Lu-ABD-PEG_4_-Lu-ABD, also referred to as [^177^Lu]Lu-Gemini. This symmetrical dimer consists of two benzyl-DOTA radiometal chelators connected by a PEG_4_ linker (**Figure 1**). This bivalent “twin” radiohapten has shown enhanced tumor retention and improved dose delivery to tumor tissue in a subcutaneous human CRC xenograft model, while maintaining comparable renal clearance to its earlier monovalent counterpart [^177^Lu]Lu-ABD 13, 19.

Here, our aim was to compare the efficacy and safety of bivalent vs. monovalent three-step DOTA-PRIT regimens in the orthotopic CRC liver metastasis model, to mimic a clinical path forward. The orthotopic CRC liver metastasis model is particularly relevant since patients with CRC commonly develop liver metastasis. While previous studies have relied primarily on subcutaneous xenograft models, studying treatment efficacy in the liver microenvironment itself may reveal different tumor behaviors, therapeutic responses, and potential resistance patterns that would not be apparent in subcutaneous xenograft models. Indeed, the selection of the best preclinical animal model research for the development of novel therapies is of tremendous importance, as the tumor microenvironment is known to influence the growth, treatment efficacy, and tumor immune response *in vivo* 21, 24. We hypothesized that the bivalent design of [^177^Lu]Lu-Gemini will enhance tumor uptake and retention in our orthotopic CRC liver metastasis model, potentially overcoming the limitations of monovalent radiohaptens in achieving curative tumor dose thresholds.

## Methods

### Cell culture and reagents

The human CRC cell lines luciferase-transduced SW1222 (SW1222-luc, GPA33^high^) and LoVo (GPA33^low^) were obtained from the Memorial Sloan Kettering Cancer Center (MSK) Ludwig Institute for Cancer Immunotherapy (New York, NY, USA). According to GPA33 quantification using Quantum Simply Cellular beads (Bangs Laboratories, Inc., Fishers, IN, USA), SW1222-luc had ~8 times higher GPA33 expression compared with LoVo (antibody binding capacity of 89023 antibodies/cell and 11342 antibodies/cell, respectively). GPA33 immunohistochemistry (IHC) confirmed these quantification findings, showing strong membranous GPA33 staining in SW1222-luc tumors compared with absent-to-low scattered GPA33 staining in LoVo tumors (**Figure S2**). Cell lines were cultured in Minimal Essential Medium (SW1222-luc) or RPMI (LoVo) supplemented with 10% heat-inactivated fetal bovine serum, 2.0 mM glutamine, 100 units/mL penicillin, and 100 units/mL streptomycin in a 37 °C environment containing 5% CO_2_. All media were purchased from the MSK Media Preparation Facility.

### Ultrasound-guided intraparenchymal liver inoculation

All animal experiments were approved by the MSK Institutional Animal Care and Use Committee (IACUC protocol: 00-03-053). For ultrasound-guided intraparenchymal liver inoculation of SW1222-luc and LoVo cell lines, the same protocol, adapted from previous studies 24, 25, was used (**Figure S3 and Supplementary Video 1**): Athymic female nude mice *Foxn1^nu^* (6-8 weeks old) were anesthetized with 3% (v/v) isoflurane gas in medical air at a flow rate of 3 L/min via an induction chamber, and then the mice were positioned supine on the ultrasound platform with a nose cone and maintained at 2% (v/v) isoflurane gas. Ultrasound gel was applied to their abdomens and the mice were imaged in B mode (2D) using a 40-MHz transducer (Vevo 2100, Visualsonics, Toronto, ON, Canada). A 30-gauge, half-inch needle mounted on a 25-μL Hamilton Luer Type syringe (Model 702 LT SYR, Hamilton, Reno, NV, USA) was aligned and advanced percutaneously into the left liver freehand using the ultrasound monitor for reference. A 20-μL suspension bolus of ~1 × 10^6^ cells/mouse in 1:1 phosphate buffer saline (PBS): Matrigel (Corning Incorporated, Corning, NY, USA) was then injected directly into the liver parenchyma at a depth of ~5 mm. The needle was slowly retracted immediately after the complete delivery of cells into the liver. The growth of the liver mass was evaluated weekly using the same anesthesia protocol, ultrasound machine, and ultrasound probe. The volume of the tumors was calculated by segmentation analysis of whole-body microCT scans (Inveon PET/CT, Siemens USA, Chicago, IL, USA) using VivoQuant software (Invicro, Boston, MA, USA). Approximately 80% of inoculated mice showed an adequately sized liver tumor (usually ranging from 200-900 mm^3^) 3 (SW1222-luc) to 4 weeks (LoVo) after intraparenchymal liver inoculation.

### Three-step DOTA-PRIT reagents

The following reagents were used for three-step DOTA-PRIT: 1) An anti-GPA33/anti-DOTA IgG-scFv BsAb (molecular weight: ~210 kDa), produced as described previously 14, 19; 2) a DOTA(Y)-conjugated poly-*N*-acetyl-galactosamine glycodendron clearing agent (molecular weight: 9059 Da), designed to remove circulating BsAbs rapidly by forming a complex via the DOTA(Y) moiety and clearing via liver asialogalactose receptor recognition and catabolism 16; and 3) a choice between two radiohaptens (**Figure 1A**) - either monovalent [^177^Lu]Lu-ABD ([^177^Lu]Lu-*S*-2-(4-aminobenzyl)-1,4,7,10-tetraazacyclododecane tetraacetic acid ([^177^Lu]Lu-ABD) or bivalent [^177^Lu]Lu-Gemini ([^177^Lu]Lu-ABD-PEG_4_-Lu-ABD). All reagents were formulated for injection in sterile saline up to a 150-μL volume and administered intravenously via the tail vein.

### Radiosynthesis of monovalent [^177^Lu]Lu-ABD and bivalent [^177^Lu]Lu-Gemini

The radiopharmaceutical [^177^Lu]LuCl_3_ (no carrier added, specific activity ~3561 GBq/mg [623 MBq/nmol]) was supplied by Isotopia Molecular Imaging (Petah Tikva, Israel). Monovalent [^177^Lu]Lu-ABD and bivalent [^177^Lu]Lu-Gemini were prepared as described previously 18, 19, 26, 27. ABD and Gemini chelated ^177^Lu efficiently, resulting in average radiochemical conversions > 99% with a molar activity of 111 MBq/nmol and 222 MBq/nmol, respectively, and a radiochemical purity > 98% for both (see the supplementary material for further details).

### *Ex vivo* biodistribution experiment

We conducted an *ex vivo* biodistribution experiment in 4 groups of mice bearing SW1222-luc tumors, to compare the monovalent [^177^Lu]Lu-ABD and bivalent [^177^Lu]Lu-Gemini radiohaptens in our three-step DOTA-PRIT approach. For each radiohapten, two different pretargeting intervals (time between the BsAb and radiohapten injections) were considered: 24 h and 48 h (**Figure 1B**). Each group (n = 20 mice) received an intravenous (i.v.) injection of anti-GPA33 BsAb (250 μg/1.19 nmol) at either t = -24 h or -48 h, and an i.v. injection of clearing agent (25 μg, 2.8 nmol) at t = -4 h, followed by an injection of monovalent [^177^Lu]Lu-ABD (3.7 MBq , 0.4 nmol) or bivalent [^177^Lu]Lu-Gemini (3.7 MBq, 0.2 nmol) at t = 0. We used half the molar amount of bivalent [^177^Lu]Lu-Gemini radiohapten compared with monovalent [^177^Lu]Lu-ABD radiohapten to normalize for the number of DOTA molecules.

**Figure 2:**
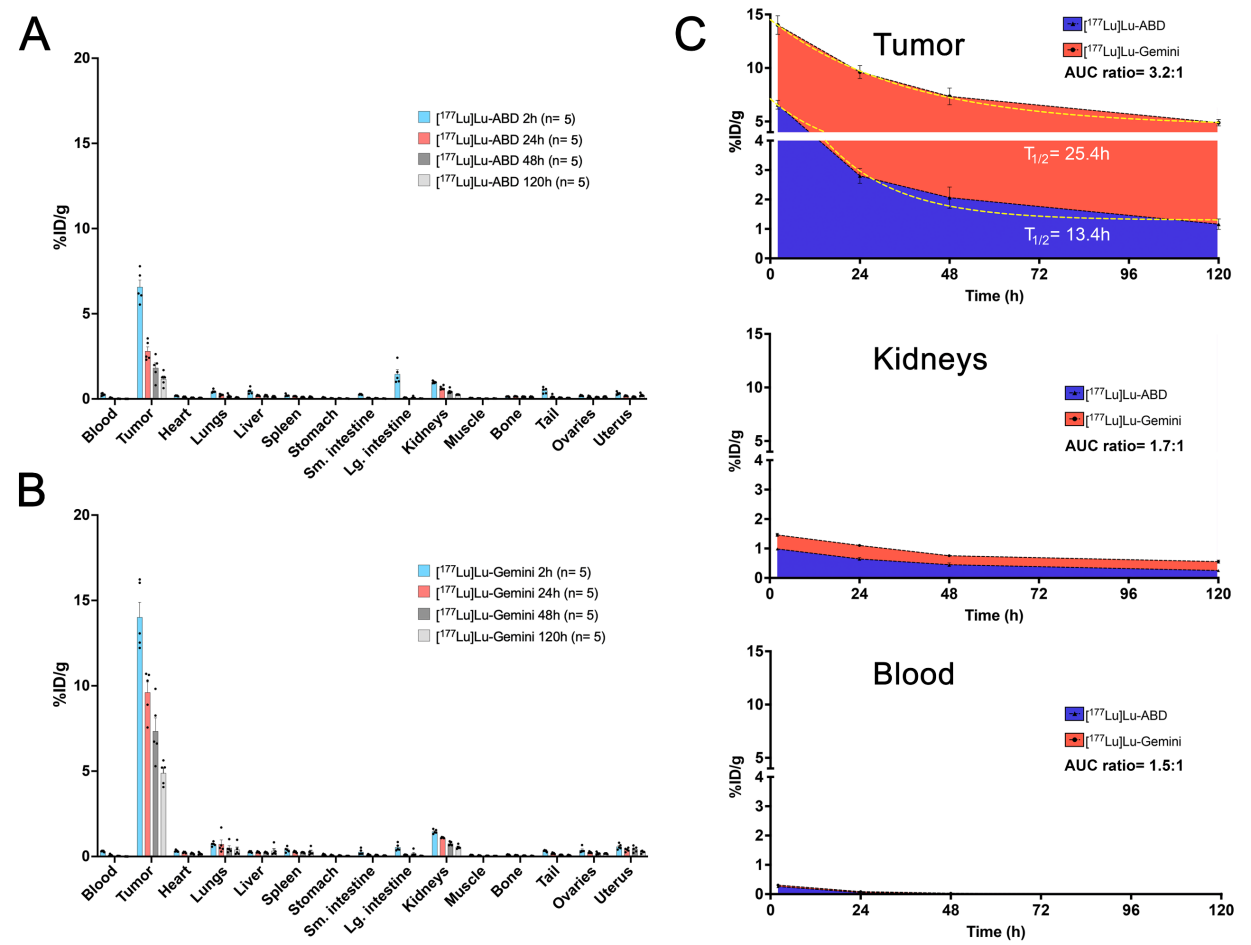
**Monovalent [^177^Lu]Lu-ABD and bivalent [^177^Lu]Lu-Gemini three-step DOTA-PRIT *ex vivo* biodistribution comparison. A.** [^177^Lu]Lu-ABD (3.7 MBq, 0.4 nmol) and **B.** [^177^Lu]Lu-Gemini (3.7 MBq, 0.2 nmol) three-step DOTA-PRIT *ex vivo* biodistribution. Values are represented as means, and error bars represent standard error of the mean (SEM). **C.** Area under the curve (AUC) analysis of *ex vivo* biodistribution for the tumor, kidneys, and blood. The longer biological half-life of [^177^Lu]Lu-Gemini on the tumor (T_1/2_ = 25.4 h) and higher plateau (4.5 %ID/g) compared with [^177^Lu]Lu-ABD (T_1/2_ = 13.4 h, plateau: 1.3 %ID/g) better aligns with the longer decay period of the therapeutic radioisotope (lutetium-177 half-life: 6.7 days), increasing tumor exposure time with a higher tumor activity retention (3.5-fold higher [^177^Lu]Lu-Gemini plateau value compared with [^177^Lu]Lu-ABD). The dashed yellow lines on the tumor graph of subfigure C represent non-linear fit one-phase decay curves for [^177^Lu]Lu-ABD (R^2^ = 0.91) and [^177^Lu]Lu-Gemini (R^2^ = 0.86) tumor biodistribution data.

At designated timepoints after the radiohapten injection (2, 24, 48, and 120 h), mice (5 per time point) were euthanized humanely by CO_2_ asphyxiation and *ex vivo* biodistribution was assessed. Tissues of interest were collected, and their activity concentration determined immediately after harvesting, using a γ-counter (Hidex Automatic Gamma Counter, Hidex Oy, Turku, Finland). Biodistribution results (**Figure 2**) were expressed as percent injected dose per gram (%ID/g) and used to estimate radiation dosimetry (see supplementary material for further details).

### SPECT and quantitative autoradiography (QAR)

SW1222-luc and LoVo tumor-bearing mice were imaged with SPECT (NanoSPECT/CT, Mediso Medical Imaging Systems, Budapest, Hungary) to compare monovalent [^177^Lu]Lu-ABD vs. bivalent [^177^Lu]Lu-Gemini tumor targeting efficacy using an established pretargeting protocol (**Figure 1B**), with an activity of 44.4 Mbq (0.4 nmol of [^177^Lu]Lu-ABD; 0.2 nmol of [^177^Lu]Lu-Gemini). We used a pretargeting interval of 24h for [^177^Lu]Lu-ABD and 48h for bivalent [^177^Lu]Lu-Gemini. Immediately after BsAb injection, long-lasting microCT contrast (ExiTron™ nano 12000, Miltenyi Biotec, Bergisch Gladbach, Germany) was administered intravenously (100 μL) via the tail vein to enhance liver tumor visualization.

**Figure 3:**
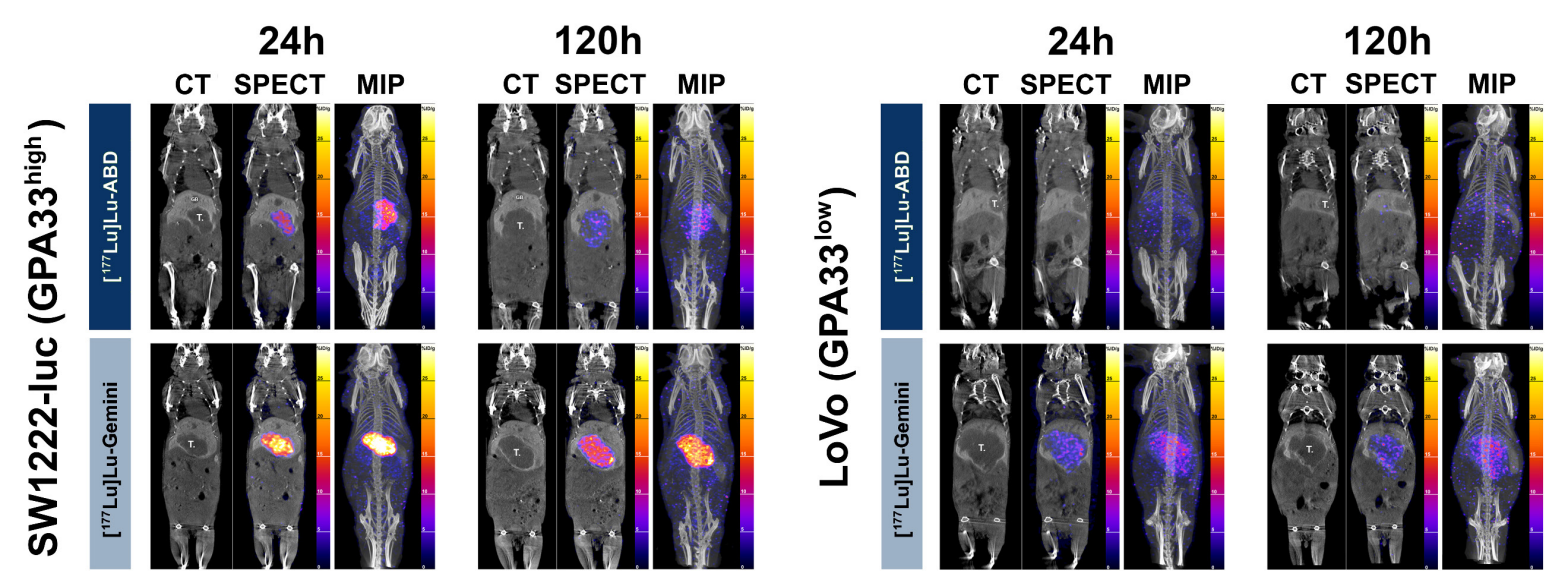
**DOTA-PRIT SPECT/CT studies with high GPA33-positive (SW1222-luc) and low GPA33-positive (LoVo) CRC liver metastasis tumor-bearing mice.** Coronal multiplanar reconstruction (MPR) and maximum intensity projection (MIP) SPECT images acquired 24 h and 120 h after 44.4 MBq injection of [^177^Lu]Lu-ABD (0.4 nmol) and [^177^Lu]Lu-Gemini (0.2 nmol). High SW1222-luc tumor uptake is noted on the 24 h SPECT images of both [^177^Lu]Lu-ABD and [^177^Lu]Lu-Gemini, with higher tumor retention of [^177^Lu]Lu-Gemini at 120 h. In LoVo tumors, superior tumor targeting and retention are noted with [^177^Lu]Lu-Gemini, compared with [^177^Lu]Lu-ABD which shows negligible tumor uptake. T.: liver tumor; GB: gallbladder; Spl.: spleen.

We imaged 5 SW1222-luc tumor-bearing mice with SPECT (**Figure 3**). Four mice were randomly selected from the DOTA-PRIT experiment: 2 from the [^177^Lu]Lu-Gemini group, 1 from the [^177^Lu]Lu-ABD group, and 1 from the control group (no BsAb injection; [^177^Lu]Lu-ABD, 44.4 MBq , 0.4 nmol). The fifth imaged mouse received monovalent [^177^Lu]Lu-ABD but was not part of the DOTA-PRIT experiment. All 5 mice were imaged at 24 h. Four of them were also re-imaged at 120 h: the 2 mice from the [^177^Lu]Lu-Gemini DOTA-PRIT group, the mouse from the [^177^Lu]Lu-ABD DOTA-PRIT group, and the mouse that received monovalent [^177^Lu]Lu-ABD but was not included in the DOTA-PRIT experiment. Additionally, one mouse from the [^177^Lu]Lu-ABD DOTA-PRIT group was re-imaged with SPECT at day 15 post treatment initiation, to assess tumor retargeting 24 h after the 3^rd^ cycle of [^177^Lu]Lu-ABD (**Figure S4**).

Of the LoVo tumor-bearing mice (n = 4) imaged with SPECT (**Figures 3 and S5**), 2 received [^177^Lu]Lu-ABD and 2 received [^177^Lu]Lu-Gemini. At 2 h, all 4 mice were imaged. At 24 h, the 2 [^177^Lu]Lu-Gemini mice and 1 mouse that received [^177^Lu]Lu-ABD were imaged. At 48 and 120 h, 1 [^177^Lu]Lu-Gemini mouse and 1 [^177^Lu]Lu-ABD mouse were imaged.

The counting rates in the reconstructed images were converted to mean %ID/g by applying a system-specific calibration factor for ^177^Lu. All SPECT images were analyzed using Vivoquant software (Invicro, Boston, MA, USA).

**Figure 4:**
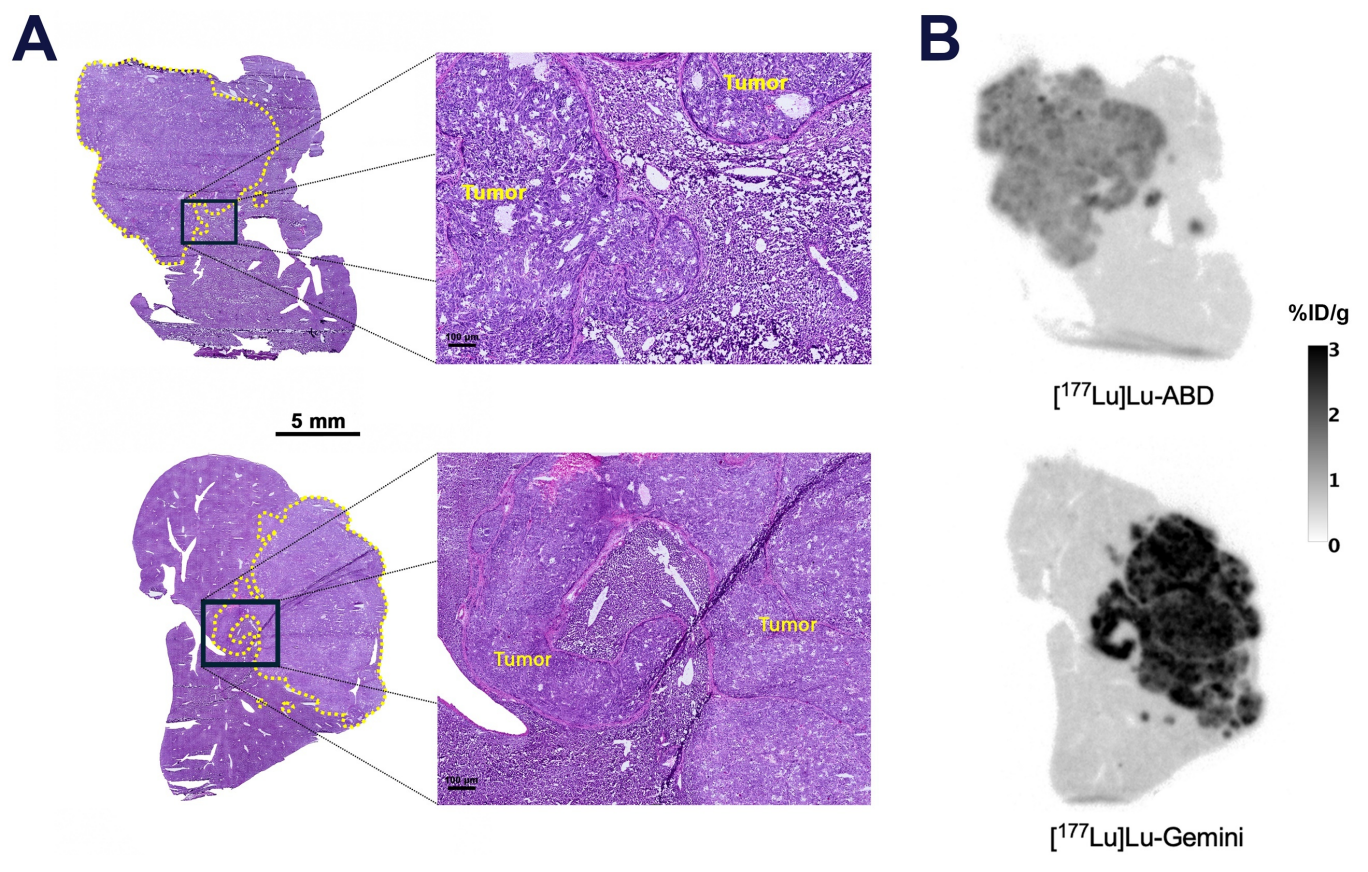
**Quantitative autoradiography (QAR). A.** Hematoxylin and eosin (H&E) micrograph of optimal cutting temperature (OCT)-frozen SW1222-luc hepatic tumors (circled in yellow), and **B.** concurrent QAR image 5 days after 44.4 MBq injection of [^177^Lu]Lu-ABD (0.4 nmol) and [^177^Lu]Lu-Gemini (0.2 nmol). Higher uptake is noted in the [^177^Lu]Lu-Gemini tumor (mean %ID/g ± SD throughout the tumor: 2.1 ± 0.4 %ID/g) compared with [^177^Lu]Lu-ABD (mean %ID/g ± SD throughout the tumor: 1.1 ± 0.2 %ID/g).

Tumor targeting efficacy of monovalent [^177^Lu]Lu-ABD (44.4 Mbq, 0.4 nmol, 24h pretargeting interval) vs. bivalent [^177^Lu]Lu-Gemini (44.4 Mbq, 0.2 nmol, 48h pretargeting interval) was also assessed in 2 additional SW1222-luc tumor-bearing mice using QAR (**Figure 4**). Mice were euthanized 5 days post monovalent or bivalent radiohapten injection, and the SW1222-luc liver tumors were resected and trimmed along with some adjacent normal hepatic parenchyma for QAR analysis.

### Three-step DOTA-PRIT experiment

**Figure 5:**
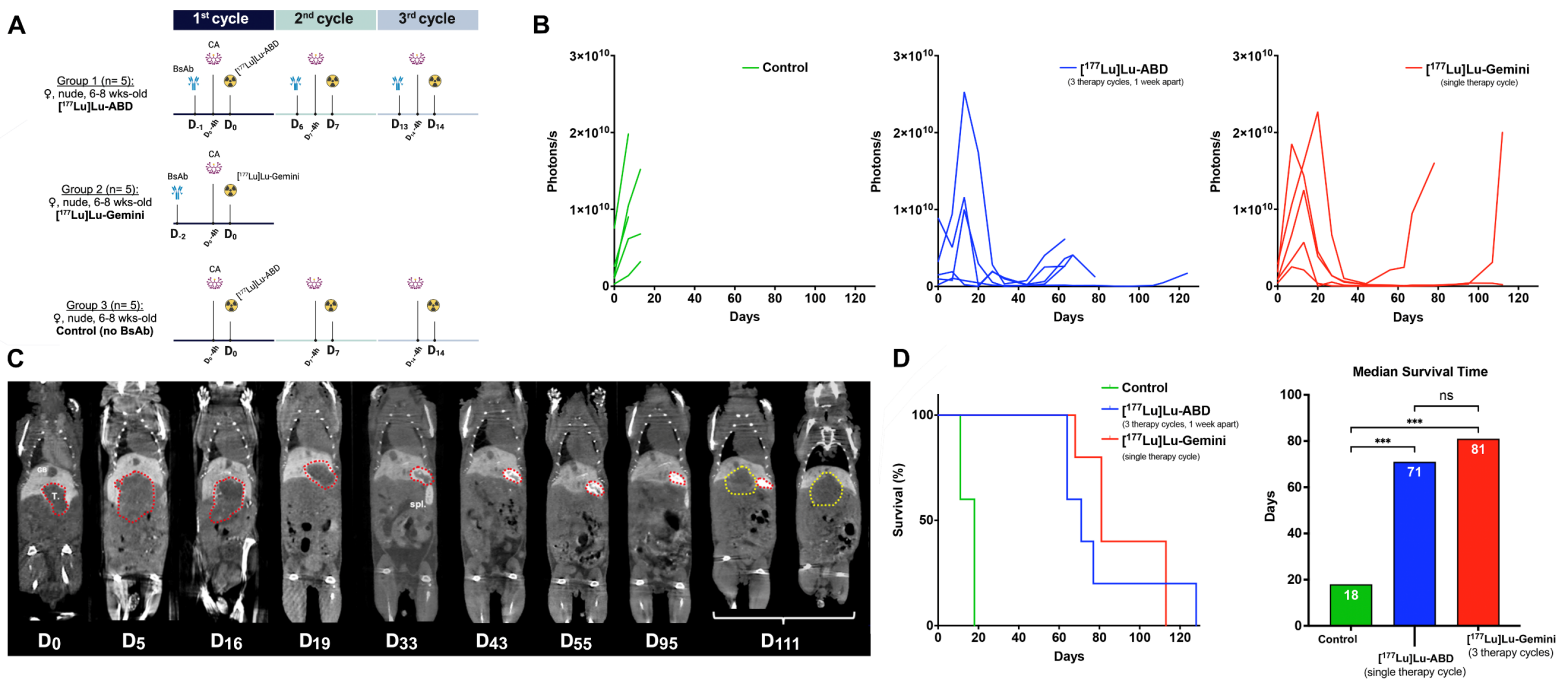
**Three-step DOTA-PRIT experiment. A.** Experimental three-step DOTA-PRIT protocol (with estimated tumor mean absorbed doses of 44 Gy and 53 Gy for 3 therapy cycles of [^177^Lu]Lu-ABD (group 1) and a single cycle of [^177^Lu]Lu-Gemini (group 2), respectively). **B.** Serial *in vivo* bioluminescence imaging (BLI). **C.** Serial coronal multiplanar reconstruction (MPR) CT images of a representative mouse treated with [^177^Lu]Lu-ABD DOTA-PRIT (same mouse as in Figure 3). The initial hepatic tumor is circled in red, and the recurrent tumor in yellow. The 2 coronal images at D_111_ have been acquired at different coronal levels. T.: tumor; GB: gallbladder; spl.: spleen. **D.** Kaplan-Meier survival curves and median survival histograms. Median survival times were compared using the log-rank (Mantel-Cox) test. ns: not statistically significant; *** p ≤ 0.001.

A DOTA-PRIT experiment was performed using three groups of SW1222-luc tumor-bearing mice (n = 5 mice per group) (**Figure 5A**). The first group (“[^177^Lu]Lu-ABD” group) underwent a monovalent [^177^Lu]Lu-ABD treatment regimen involving three treatment cycles one week apart, with anti-GPA33 BsAb (250 μg/1.19 nmol) at t = -24 h, clearing agent (25 μg, 2.8 nmol) at t = -4 h, and monovalent [^177^Lu]Lu-ABD (44.4 MBq , 0.4 nmol) at t = 0 h (24 h pretargeting interval). The second group (“[^177^Lu]Lu-Gemini” group) underwent a bivalent [^177^Lu]Lu-Gemini treatment regimen involving only a single cycle, with anti-GPA33 BsAb (250 μg/1.19 nmol) at t = -48 h, clearing agent (25 μg/2.8 nmol) at t = -4 h, and bivalent [^177^Lu]Lu-Gemini (44.4 MBq , 0.2 nmol) at t = 0 h (48 h pretargeting interval). The third group was a control group, with all mice undergoing a multicycle treatment (three treatment cycles one week apart) of only clearing agent at t = -4h and monovalent [^177^Lu]Lu-ABD (44.4 MBq, 0.4 nmol) at t = 0 h, without anti-GPA33 BsAb. Immediately after the BsAb injection, long-lasting microCT contrast (ExiTron™ nano 12000, Miltenyi Biotec, Bergisch Gladbach, Germany) was administered intravenously (100 μL) via the tail vein to enhance liver tumor visualization.

Periodic measurements of body weight and hematological parameters were conducted to evaluate potential signs of toxicity. Tumor progression was monitored with bioluminescence imaging (BLI) using the IVIS Spectrum *In Vivo* Imaging System (PerkinElmer, Inc., Waltham, MA, USA) and microCT using the Inveon PET/CT system (Siemens USA, Chicago, IL, USA).

Mice were sacrificed if their tumor volume exceeded 1500 mm^3^, if they were lethargic and/or presented a distended abdomen or other significant clinical signs, or if excessive weight loss (20%) from their pretreatment baseline weight was noted. Complete or partial necropsies were performed in selected mice depending on pathology service availability, along with histopathology and GPA33 IHC. GPA33 IHC staining positivity was evaluated subjectively and graded as a percentage of positively stained cells in the tissue samples.

### Data analysis

Quantitative data were expressed as mean ± standard error of the mean (SEM) unless otherwise noted. Kaplan-Meier survival curves were analyzed using the Mantel-Cox test. For comparisons between groups, multiple Mann-Whitney U tests were performed with p-values adjusted for multiple comparisons using the two-stage step-up method of Benjamini, Krieger, and Yekutieli with a target false discovery rate (FDR) of 5%. All statistical analyses were performed using Prism 10 (GraphPad Software, San Diego, CA, USA). For all studies, a p-value less than 0.05 was considered statistically significant.

## Results

### *Ex vivo* biodistribution in tumor-bearing mice and dosimetry

Both monovalent [^177^Lu]Lu-ABD and bivalent [^177^Lu]Lu-Gemini radiohaptens showed rapid and effective SW1222-luc tumor targeting (**Figures S6 and Table S1**). [¹⁷⁷Lu]Lu-Gemini however achieved significantly higher tumor uptake (%ID/g) compared with [¹⁷⁷Lu]Lu-ABD across all evaluated time points (2 h, 24 h, 48 h, and 120 h) for both 24 h and 48 h pretargeting intervals. There was no statistical difference in tumor uptake between the 24 h and 48 h pretargeting intervals for [¹⁷⁷Lu]Lu-Gemini, while the 24 h pretargeting interval for [¹⁷⁷Lu]Lu-ABD consistently resulted in higher tumor uptake compared with the 48 h interval at all time points (**Figure S6**).

Tumor biodistribution data of [^177^Lu]Lu-ABD (24 h pretargeting interval) and [^177^Lu]Lu-Gemini (48 h pretargeting interval) were analyzed using a non-linear one-phase decay curve-fitting model (**Figure 2C**), with successful fitting of both curves (R² = 0.91 for [^177^Lu]Lu-ABD, and 0.86 for [^177^Lu]Lu-Gemini). The analysis confirmed the superior tumor retention of [^177^Lu]Lu-Gemini (48 h pretargeting) compared with [^177^Lu]Lu-ABD (24 h pretargeting), with a tumor biological half-life and plateau of respectively 13.4 h and 1.3 %ID/g for [^177^Lu]Lu-ABD, and 25.4 h and 4.5 %ID/g for [^177^Lu]Lu-Gemini. The extended biological tumor half-life of [^177^Lu]Lu-Gemini better complemented lutetium-177 physical half-life (6.7 days), while its elevated plateau provided a 3.5-fold increase in sustained tumor activity compared to [^177^Lu]Lu-ABD - both characteristics having the potential of significantly improving the therapeutic efficacy of [^177^Lu]Lu-Gemini.

Mean absorbed doses for tumor and normal tissues (**Table 1**) were estimated for both radiohaptens based on *ex vivo* biodistribution results (**Table S1**), to help guide our DOTA-PRIT experiment and predict dose-limiting tissues. The therapeutic index (TI) values, representing the ratio between the tumor and the respective normal tissue mean absorbed doses were also calculated, with higher TI values indicating better therapeutic efficacy with potentially fewer side effects. For 24 h pretargeting interval [^177^Lu]Lu-ABD, the mean absorbed doses (in cGy/MBq) and TI were as follows: tumor 32.88, blood 2.14 (TI = 15), normal liver 3.14 (TI = 10), and kidneys 6.94 (TI = 5). In comparison, 48 h pretargeting interval [^177^Lu]Lu-Gemini showed higher mean absorbed doses (in cGy/MBq): 119.88 for tumor, 2.40 for blood (TI = 50), 5.85 for normal liver (TI = 20), and 13.23 for kidneys (TI = 9). Based on these estimates, [^177^Lu]Lu-Gemini PRIT would deliver a significantly higher mean absorbed dose to the tumor (by a factor of ~4) and achieve more favorable TIs compared with monovalent [^177^Lu]Lu-ABD PRIT across the evaluated tissues. For both radiohaptens, the kidneys would receive the highest dose compared with other organs.

When considering the administered activity per cycle in our DOTA-PRIT experiment (44.4 MBq), both [^177^Lu]Lu-ABD and [^177^Lu]Lu-Gemini treatment regimens would lead to significant tumor responses (estimated cumulative absorbed dose delivered to the tumor: 44 Gy for the [^177^Lu]Lu-ABD treatment regimen (three cycles) and 53 Gy for the [^177^Lu]Lu-Gemini treatment regimen (single cycle)) while maintaining kidney doses below the currently accepted normal tissue dose limit and toxicity endpoint of 23-26 Gy for radiopharmaceutical therapy 28 (projected cumulative doses of 9.2 Gy for the [^177^Lu]Lu-ABD treatment regimen (three cycles) and 5.9 Gy for the [^177^Lu]Lu-Gemini treatment regimen (single cycle)). This tumor dose comparison underscores the tremendous advantage of the single-cycle [^177^Lu]Lu-Gemini treatment regimen which allows, with just one administration, the delivery of an estimated tumor dose surpassing the cumulative dose achieved by the [^177^Lu]Lu-ABD treatment regimen over three treatment cycles.

Using the activity concentration in the blood as a surrogate for bone marrow activity concentration, our estimates also indicate that the mean absorbed dose to the bone marrow, considered the most radiosensitive organ, would remain below the threshold of 2.5 Gy for the [^177^Lu]Lu-Gemini treatment regimen (1.1 Gy), while this threshold would be reached after three treatment cycles of the [^177^Lu]Lu-ABD treatment regimen (2.8 Gy over three cycles) 28. It is, however, important to note that the bone marrow can regenerate to some extent during multicycle treatments, allowing for potential retreatment without the need for extended recovery periods, unlike slower regenerating organs such as the liver or kidneys, where radiation effects are viewed as cumulative and additive toward an upper limit 28.

### SPECT and QAR results

SPECT results at 24 h showed efficient tumor targeting by both [^177^Lu]Lu-ABD and [^177^Lu]Lu-Gemini for SW1222-luc tumors (**Figure 3**). With [^177^Lu]Lu-ABD, the tumor uptake was 5.39 ± 1.34 %ID/g (n = 2), and the tumor-to-normal tissue (T:NT) ratios were 15.8 (kidneys) and 45.9 (blood). With [^177^Lu]Lu-Gemini, the tumor uptake was 15.23 ± 2.46 %ID/g (n = 2), and the T:NT ratios were 23.7 (kidneys) and 61.0 (blood). Meanwhile, at this same timepoint, negligible tumor targeting, with a tumor uptake of 0.07 %ID/g (image not shown), was observed in the imaged control mouse. Of note, at 5 days, follow-up SPECT (**Figure 3**) and QAR (**Figure 4**) showed superior tumor retention of [^177^Lu]Lu-Gemini (9.44 ± 1.00 %ID/g, n = 2, on SPECT analysis) compared to [^177^Lu]Lu-ABD (1.76 %ID/g, n = 1, on SPECT analysis). Efficient tumor retargeting after the 3^rd^ cycle of [^177^Lu]Lu-ABD was also confirmed on SPECT (**Figure S4**), with a tumor uptake of 6.95 %ID/g at 24 h.

The observed discrepancies in SW1222-luc tumor uptake values between the *ex vivo* biodistribution experiment and SPECT imaging (for instance, 9.62 ± 0.55 %ID/g (n= 5) at 24 h for [¹⁷⁷Lu]Lu-Gemini in the *ex vivo* biodistribution experiment, versus 15.23 ± 2.46 %ID/g (n= 2) with SPECT imaging) could be attributed to several factors. Firstly, the smaller SPECT imaging sample size likely played an important role, with a notable individual variation in tumor uptake, as demonstrated by the high SEM on the SPECT images compared with the *ex vivo* biodistribution experiment. Secondly, the quantification could be affected by the inherent methodological variations between the two techniques and how regions of interest (ROIs) are defined and analyzed on SPECT images for %ID/g calculations versus direct tissue measurements in the *ex vivo* biodistribution experiment.

Finally, SPECT results at 24 h showed superior tumor targeting by [^177^Lu]Lu-Gemini compared with [^177^Lu]Lu-ABD for low GPA33-positive LoVo tumors (**Figures 3 and S5**). With [^177^Lu]Lu-Gemini, the tumor uptake was 2.91 ± 0.23 %ID/g (n = 2), and the T:NT ratios were 4.4 (kidneys) and 6.7 (blood). In contrast, with [^177^Lu]Lu-ABD, the tumor uptake was minimal at only 0.57 %ID/g (n = 1), and the T:NT ratios were 1.0 (kidneys) and 3.4 (blood). At 120 h, LoVo tumor uptake remained relatively unchanged for both radiohaptens. With [^177^Lu]Lu-Gemini, the tumor uptake was 2.69 %ID/g (n = 1), with minimally improved T:NT ratios (5.8 for kidneys, 10 for blood). With [^177^Lu]Lu-ABD, the tumor uptake remained minimal at 0.45 %ID/g (n = 1), and the T:NT ratios were 1.8 (kidneys) and 3.3 (blood).

### DOTA-PRIT experiment results

The liver tumor response curves (CT and BLI) for treatment and control (i.e., clearing agent and [^177^Lu]Lu-ABD but no BsAb) groups are shown in **Figures 5 and S7**. In the control group, rapid tumor progression above the experiment endpoint (> 1500 mm^3^) was observed, leading to the euthanasia of all mice (n = 5) by day 18. In [^177^Lu]Lu-ABD and [^177^Lu]Lu-Gemini treatment groups, the liver tumors continued to grow for 2-3 weeks after the initiation of the 1^st^ treatment cycle, before decreasing in size and BLI signal, reaching a nadir at ~45 days (**Figures 5 and S7**). At this timepoint, only residual liver tumors/scar tissue surrounded by a rim of contrast medium were visible on CT (**Figure 5C**). Liver tumor size decreased from 153 ± 92 mm^3^ at D_0_ to 18 ± 8 mm^3^ at D_44_ for the [^177^Lu]Lu-ABD group (n = 2 mice imaged), and from 434 ± 174 mm^3^ to 60 ± 20 mm^3^ for the [^177^Lu]Lu-Gemini group (n = 5 mice imaged). 3/5 mice from the [^177^Lu]Lu-ABD group had perivenous extravasation of contrast medium (visible on CT, not shown), which prevented the evaluation of their liver tumor size on CT. To minimize additional handling of the treated mice, a second injection of contrast medium was not attempted. Injecting the contrast medium at a separate timepoint (t = -72 h for instance) rather than immediately after the BsAb could prevent this issue and should be considered in future studies. On BLI, the total photon flux decreased 10-fold for both treatment groups, from 2.97 × 10^9^ ± 1.56 × 10^9^ photons/s at D_0_ to 2.95 × 10^8^ ± 1.18 ×10^8^ photons/s at D_44_ for the [^177^Lu]Lu-ABD group (n = 5 mice imaged), and from 1.41 × 10^9^ ± 4.46 × 10^8^ photons/s to 1.56 × 10^8^ ± 5.77 × 10^7^ photons/s for the [^177^Lu]Lu-Gemini group (n = 5 mice imaged). Treatments were well tolerated, without significant weight loss or hematologic changes between groups or deviation from reference ranges (**Figure S8**).

Eventually, all mice from the treatment groups met one of the experiment endpoint criteria, requiring euthanasia. For the three [^177^Lu]Lu-ABD-treated mice with contrast medium perivenous extravasation and for the control mice that did not receive contrast medium, the decision to euthanize was taken when significant abdominal distension/tumor burden was detected on abdominal palpation (tumors reaching a diameter of ~1.4 cm, corresponding to an approximate volume of 1500 mm^3^). The median survival time (**Figure 5D**) was 18 days for the control group, 71 days for the [^177^Lu]Lu-ABD group, and 81 days for the [^177^Lu]Lu-Gemini group - with survival time being statistically different between treatment and control groups (p < 0.01, logrank Mantel-Cox), but not between treatment groups (p = 0.53, log-rank Mantel-Cox).

### Toxicity assessment

At the time of death, 8/10 treated mice exhibited large recurrent liver tumors exceeding the experiment endpoint size (1500 mm^3^), requiring euthanasia. Necropsies were performed on 7/10 treated mice, some undergoing complete examinations (5/7 necropsied mice), and others partial necropsies (2/7 necropsied mice), limited to the liver, kidneys, and femur/bone marrow (**Table S2**), depending on pathology service availability. Histopathological examination revealed large liver adenocarcinomas consistent with SW1222-luc xenograft implantation in 5/7 necropsied treated mice, often with metastases to abdominal structures, and in one case, to the lungs. Two treated mice demonstrated distinct outcomes, i.e., not presenting with a distended abdomen and/or a recurrent liver mass (**Figure S9**). One [^177^Lu]Lu-Gemini-treated mouse (G2M4 mouse in **Table 2**) had complete liver tumor regression, with only scar tissue identified histologically at the inoculation site, consistent with complete response to treatment (**Figure S9A-C**). This mouse, however, developed a head tilt at day 81, requiring euthanasia. There was no detectable neoplasia in the tissues submitted for histology (limited to liver, kidneys, and femur/bone marrow), but a full necropsy was not performed, and the possibility of a distant metastasis could therefore not be excluded (**Table S2**). Another [^177^Lu]Lu-Gemini-treated mouse (G2M5 mouse in **Table 2**), euthanized for acute hindlimb paralysis on day 68, had a residual liver tumor mostly composed of fibrosis, necrotic debris, mucin, and granulomatous inflammation, with only few viable tumor cells (**Figure S9D-G**). This mouse also developed a large highly cellular spinal cord metastasis, leading to euthanasia (**Figure S9H-K**).

Necropsies (2 complete and 3 partial) were also performed on control mice. A consistent histological finding among treated mice compared with control mice was marked ovarian atrophy (**Table S2**), absent in control mice, aligning with previous studies and interpreted as a treatment-related effect 19, 29, 30. No significant microscopic change indicative of treatment-related radiotoxicity was observed in the kidneys, urinary bladder, liver, spleen, or bone marrow. One [^177^Lu]Lu-Gemini-treated mouse displayed moderate chronic cholangiohepatitis and occasional foci of hepatocyte necrosis, but similar liver necrosis was observed in control mice and was therefore attributed to the large liver tumors rather than related to treatment. Additional microscopic findings (**Table S2**) were considered incidental or typical background changes for the mouse strain, sex, and age, and were not interpreted as treatment-related effects 31.

### GPA33 IHC

**Figure 6:**
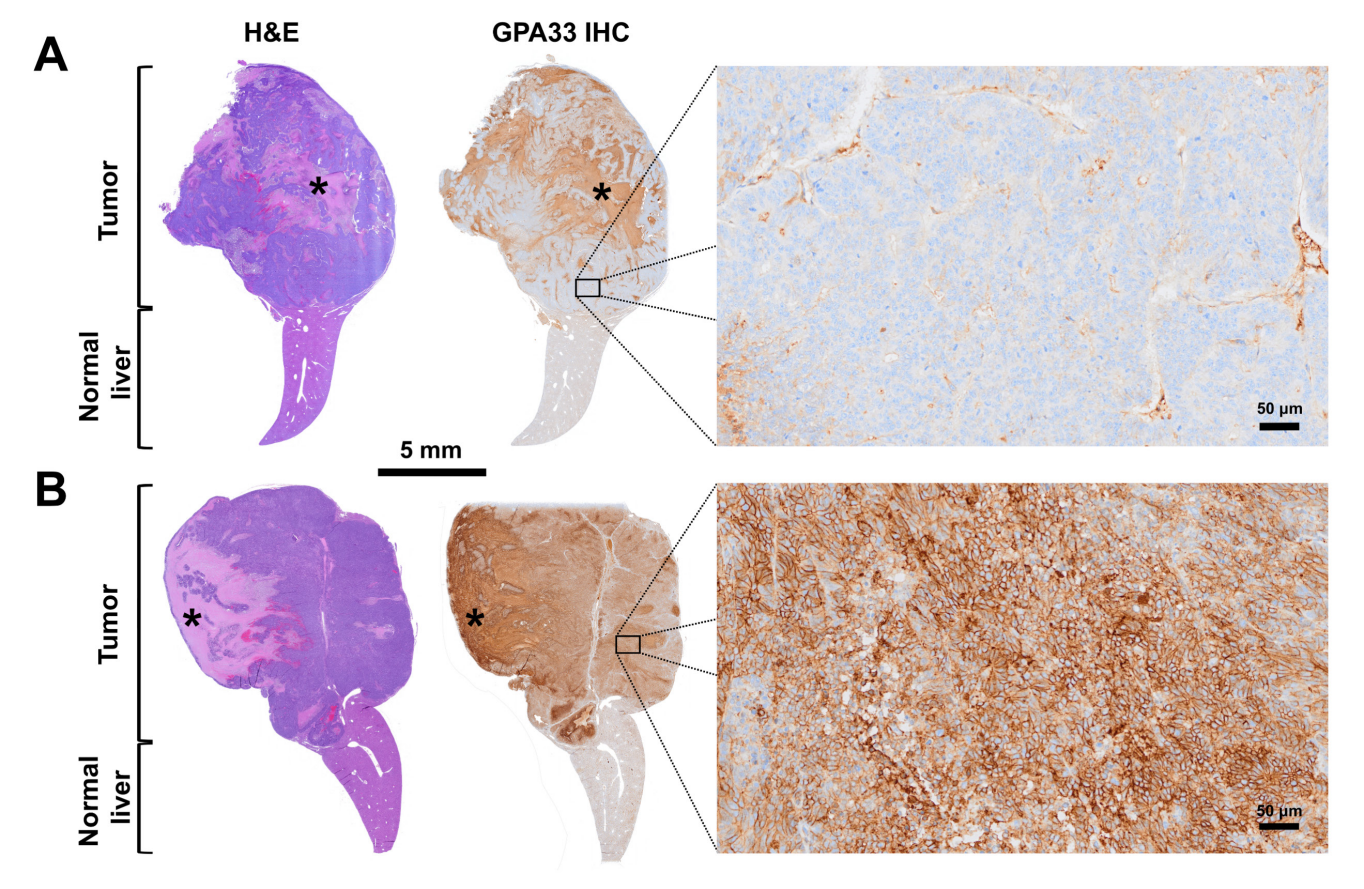
**GPA33 differential expression in recurrent liver tumors post-three-step DOTA-PRIT** (experimental three-step DOTA-PRIT protocol in Figure 5A).** A.** Representative hematoxylin and eosin (H&E) micrograph (left) and GPA33 immunostaining (right) of a recurrent GPA33(-) liver tumor 64 days post [^177^Lu]Lu-ABD three-step DOTA-PRIT (G1M2 in Table 2). High magnification on viable cellular areas of the tumor shows negligible membranous GPA33 expression (5% immunohistochemistry (IHC) signal positivity). **B.** Representative H&E micrograph (left) and GPA33 immunostaining (right) of a recurrent GPA33(+) hepatic tumor 113 days post-[^177^Lu]Lu-Gemini three-step DOTA-PRIT (G2M3 in Table 2). High magnification on viable cellular areas of the tumor shows diffuse and strong, membranous GPA33 expression (90% IHC signal positivity). Large necrotic areas, which show non-specific staining on IHC, are marked with an asterisk.

GPA33 immunostaining was performed on the recurrent liver tumors from 5 treated mice, the liver of the 2 treated mice without a recurrent liver tumor, and the liver from 1 control mouse (**Figure S9**). In addition, GPA33 immunostaining was also performed on 6 extrahepatic metastatic lesions (pancreas, small intestine, spinal cord, mesometrium, axillary lymph node, and diaphragm) from 3 treated mice (2 from the [^177^Lu]Lu-Gemini group and 1 from the [^177^Lu]Lu-ABD group) (**Table 2 and Figure S10**). Among the 12 viable tumor samples from treated mice (excluding the liver of the mouse with only scar tissue (G2M4 in **Table 2**)), 83% (10/12) demonstrated high GPA33 cell positivity, with over 70% of tumor cells staining positively (**Table 2 and Figure S10A-G**). However, 17% (2/12) of the samples had less than 20% positively stained tumor cells (**Figure 6, Figure S9H-K, and Table 2**), suggesting the possibility of immune tumor evasion and/or loss of GPA33 antigen/epitope expression in these samples. Strong GPA33 IHC signal positivity was present in the liver tumor of the control mouse (image not shown).

## Discussion

This study demonstrates the considerable potential of three-step DOTA-PRIT to treat CRC liver metastases, particularly three-step DOTA-PRIT based on the bivalent [^177^Lu]Lu-Gemini radiohapten, as demonstrated in an orthotopic CRC liver metastasis model. Tumor response to DOTA-PRIT was assessed using a combination of sequential CT scans and BLI in five mice that underwent the monovalent [^177^Lu]Lu-ABD treatment regimen (three weekly treatment cycles), five mice that underwent the bivalent [^177^Lu]Lu-Gemini treatment regimen (single-cycle treatment), and five control mice that received monovalent [^177^Lu]Lu-ABD but did not have any BsAb injection. The control group had the lowest median survival of 18 days, with progressive increase tumor size requiring sacrifice of all control mice by day 18.

In comparison, both the [^177^Lu]Lu-ABD and [^177^Lu]Lu-Gemini treatment regimens resulted in remarkable SW1222-luc tumor targeting efficacy, which ultimately led to a 4-fold increase in survival compared with control group, without evidence of acute toxicity. Of note, median survival was similar for mice treated with multicycle [^177^Lu]Lu-ABD (71 days) and those treated with single-cycle [^177^Lu]Lu-Gemini (81 days).

For our DOTA-PRIT experiment, the pretargeting interval for each radiohapten was selected based on previous publications 19, 27, 32 and the results of our *ex vivo* biodistribution experiment. For [¹⁷⁷Lu]Lu-ABD, using a 24 h pretargeting interval was justified by the consistently higher tumor uptake at all time points compared to the 48 h pretargeting interval (**Figure S6**), along with favorable TI (**Table 1**) for all examined organs. For [¹⁷⁷Lu]Lu-Gemini, although tumor, kidney or blood uptake did no show statistically significant differences between the two pretargeting intervals (**Figure S6**), we chose the 48 h interval because of its improved TI (**Table 1**), and to align with recent studies in which longer intervals (up to 3 days) have been used to optimize tumor targeting and blood clearance 13, 29, 33. Using these pretargeting intervals, comparable radiation doses to tumors were also achieved with both radiohaptens: 44 Gy for 3 therapy cycles of 44.4 Mbq of [¹⁷⁷Lu]Lu-ABD, and 53 Gy for a single cycle of 44.4 Mbq of [¹⁷⁷Lu]Lu-Gemini, allowing adequate therapeutic efficacy comparison of the two platforms.

Both treatment regimens maintained high TIs for blood and kidney, with [^177^Lu]Lu-Gemini exhibiting superior performance by delivering a mean absorbed dose to the tumor almost four times higher compared with [^177^Lu]Lu-ABD and achieving more favorable therapeutic indices across the evaluated tissues (**Table 1**). Finally, a maximum tolerated dose was not reached for either treatment regimen.

The advantages of the [^177^Lu]Lu-Gemini over the [^177^Lu]Lu-ABD were even more noticeable in low GPA33-positive LoVo tumors. In these tumors, the tumor uptake of [^177^Lu]Lu-Gemini on SPECT images acquired 24 h and 120 h post injection was clearly higher than that of [^177^Lu]Lu-ABD ([^177^Lu]Lu-Gemini: 2.91 ± 0.32 %ID/g (n = 2) and 2.69 %ID/g (n = 1) at 24 h and 120 h, respectively; [^177^Lu]Lu-ABD: 0.57 %ID/g (n = 1) and 0.45 %ID/g for ABD (n = 1), at 24 h and 120 h, respectively). These results confirmed the superiority of the [^177^Lu]Lu-Gemini treatment regimen over the [^177^Lu]Lu-ABD treatment regimen, which is attributed to [^177^Lu]Lu-Gemini's dual binding feature and higher tumoral retention over time. This finding could have a tremendous clinical impact: by allowing similar or even higher tumor targeting while decreasing the number of drug injections, patients' treatment burden can be improved. On the other hand, the increased renal uptake observed with [^177^Lu]Lu-Gemini warrants careful consideration for future clinical translation, especially if multicycle [^177^Lu]Lu-Gemini treatments will be considered. Similarly, even though our dosimetry estimates indicate that a single dose of [^177^Lu]Lu-Gemini would keep the bone marrow mean absorbed dose (1.1 Gy) below the accepted dose limit of 2-3 Gy, multicycle [^177^Lu]Lu-Gemini treatments could significantly increase this dose, requiring careful monitoring for hematological toxicity.

Our GPA33 IHC results highlight important considerations for three-step DOTA-PRIT clinical application. While most recurrent liver tumors and extrahepatic metastases (i.e., metastases beyond the liver) maintained high GPA33 expression post-treatment, a small subset (17%, 2/12) of submitted samples showed less than 20% positively stained tumor cells (**Figures 6 and S9**). This result suggests possible immune tumor evasion and/or loss of GPA33 antigen/epitope expression. Potential mechanisms may include the removal of antigens from the cell surface (via internalization or trogocytosis), disrupted trafficking to the cell membrane, transcriptional downregulation, or genomic alterations 34, 35. A reduction in the average expression of the targeted antigen after treatment could also occur due to the elimination of tumor cell subpopulations with the highest antigen levels 34, 35. This finding emphasizes the importance of the theranostic concept, i.e., the necessity of conducting immunoPET with a suitable companion PET agent before considering PRIT (in our three-step DOTA-PRIT approach, for instance, the β^+^ isotope yttrium-86 could be used as an imaging surrogate for lutetium-177). Finally, this finding accentuates the potential benefits of multivalent antigen targeting, either simultaneously or sequentially, and the selection of different antigen combinations (depending on antigen density differences across tumors) to overcome tumor treatment escape.

Our current orthotopic CRC liver SW1222-luc metastasis model complements previous DOTA-PRIT results obtained with a subcutaneous SW1222 xenograft model 19. While both models confirmed the superiority of [¹⁷⁷Lu]Lu-Gemini over [¹⁷⁷Lu]Lu-ABD, there were notable differences in tumor *ex vivo* biodistribution results and therapy outcomes. In the biodistribution experiment, [^177^Lu]Lu-Gemini tumor %ID/g was higher in the subcutaneous model compared with the orthotopic liver model. For instance, at 2 h [¹⁷⁷Lu]Lu-Gemini (48 h pretargeting) tumor uptake was 29.5 ± 6.0 %ID/g (mean ± SD, n= 4) in the subcutaneous model and 14.0 ± 1.9 %ID/g (mean ± SD, n= 5) in the liver model. At 120 h, it was 16.7 ± 3.0 %ID/g (mean ± SD, n= 4) in the subcutaneous model and 4.9 ± 0.7 %ID/g (mean ± SD, n= 5) in the orthotopic liver model. This led to much higher estimated mean absorbed doses to the tumor and TI in the subcutaneous model: 455 cGy/MBq for [¹⁷⁷Lu]Lu-Gemini and 69.79 cGy/MBq for [¹⁷⁷Lu]Lu-ABD, versus 119.88 cGy/MBq for [¹⁷⁷Lu]Lu-Gemini and 32.88 cGy/MBq for [¹⁷⁷Lu]Lu-ABD in the orthotopic liver model. [¹⁷⁷Lu]Lu-Gemini therapy in the subcutaneous model also achieved 100% complete response with 40% histologic cure using a single 44.4 MBq treatment, while all treated mice in the orthotopic liver model developed a recurring liver mass and/or metastatic disease ultimately requiring euthanasia. The tumor microenvironment and metastatic potential of the hepatic tumors likely influenced therapeutic agent accessibility and radiation response, which could partially explain these differences. The survival difference between the 2 models may also relate to the shorter duration of the subcutaneous model experiment (80 days, versus 128 days for our current liver model). One of the most significant differences that also likely contributed to this survival discrepancy was the CRC extrahepatic dissemination in the orthotopic liver model (**Table S2**), which was absent in the subcutaneous model, where tumors remained confined to the injection site 19. While this could be due to true hematogenous metastatic dissemination related to hepatic tumor localization, it may also have resulted from inadvertent direct hematogenous systemic seeding into the hepatic venous circulation at the time of inoculation. This assumption is supported by the presence of extrahepatic CRC dissemination (**Table S2**: right ventricle, lungs, abdominal wall, mesometrium) in 2 fully necropsied control mice, despite their short survival time (11 and 18 days). Because of the small size of the mouse liver and dense parenchymal vascularization, such dissemination may however be difficult to avoid. To prevent extrahepatic cell migration, we have refined our current ultrasound-guided intraparenchymal liver inoculation protocol, to inject even smaller inoculation volumes (~10 μL, 2-3 million cells) in 100% Matrigel.

In our PRIT experiment, marked ovarian atrophy was observed in both therapy groups, aligning with our previous preclinical [^177^Lu]Lu-PRIT results 19, 29, 30 and suggesting radiation-induced damage to germ cells. The close anatomical proximity of the ovaries to the kidneys in mice compared to humans, along with their distinct body size and conformation, could have also contributed to the ovarian atrophy observed in our PRIT preclinical models. While the estimated median lethal dose (LD50) for ovarian follicles is 4 Gy in humans 36, 37, a dose higher than 20 Gy in women below 40 years of age and 6 Gy in older women is required to induce permanent ovarian failure 28, 36, 38, 39 and our estimated doses to the ovaries remained way below these critical thresholds (1.6 Gy for a single therapy cycle of [^177^Lu]Lu-Gemini and 3.0 Gy for 3 cycles of [^177^Lu]Lu-ABD). Unlike ovaries where the main concern is sterilization, uterine radiation damage is linked to a functional capacity for pregnancy and specific dose limits are not as confidently established. A uterine radiation dose below 4 Gy should however not impair pregnancy function 40. In our study, the [^177^Lu]Lu-ABD cumulative dose (5.4 Gy for 3 therapy cycles) slightly exceeded this level, while [^177^Lu]Lu-Gemini (2.7 Gy) fell below it. Nevertheless, both estimated doses remained well under the suggested critical thresholds for pregnancy concerns (uterine dose > 45 Gy during adulthood and > 25 Gy in childhood 40), inferring that both PRIT platforms should preserve uterine functional capacity for pregnancy. These findings however warrant further investigation and highlight the importance of comprehensive reproductive toxicity assessment when developing radiopharmaceutical therapies.

One of the major limitations of our current 3-step DOTA-PRIT regimen is the necessity of a clearing agent to remove unbound BsAbs. To address this, we have recently established a 2-step approach alleviating the need for clearing agent, by engineering a dual scFv bispecific platform (anti-GPA33/anti-DOTA) fused to a human p53 tetramerization (p53_tet_)-based self-assembling and disassembling (SADA) domain 33, 41. Efforts are currently ongoing to combine this SADA BsAb platform with [^177^Lu]Lu-Gemini. Preliminary investigation of [^177^Lu]Lu-Gemini in a subcutaneous xenograft model has shown promising results 33, and we have also successfully established a pretargeting PET approach using [^86^Y]Y-Gemini as an imaging surrogate of [^177^Lu]Lu-Gemini (unpublished data). Upcoming studies are currently in progress to evaluate the [^177^Lu]Lu-Gemini SADA-based PRIT and PET approach in our orthotopic CRC liver metastasis model.

## Conclusions

Three-step DOTA-PRIT, particularly three-step DOTA-PRIT based on the bivalent [^177^Lu]Lu-Gemini radiohapten, was effective in treating CRC liver metastasis in an orthotopic CRC liver metastasis model. Both monovalent and bivalent three-step DOTA-PRIT regimens increased the median survival 4-fold compared with controls and did not exert any acute toxicity. Notably, tumor dose efficiency was markedly enhanced with the bivalent [^177^Lu]Lu-Gemini system, which delivered an estimated mean absorbed dose to the tumor of 53 Gy in a single cycle (119.88 cGy/MBq), exceeding the 44 Gy cumulative absorbed dose achieved through three therapy cycles of [^177^Lu]Lu-ABD (32.88 cGy/MBq per cycle). The enhanced tumor uptake and retention of bivalent [^177^Lu]Lu-Gemini could therefore be of tremendous benefit in the clinic. Our results also highlight the importance of a theranostic approach and the potential need for multivalent targeting strategies to address tumor heterogeneity and the possible loss of tumor antigen/selective tumoral cells subpopulation during serial treatments.

## Supplementary Material

Supplementary figures and tables.

Supplementary Video 1.

## Figures and Tables

**Table 1:** Three-step DOTA-PRIT estimated mean absorbed doses and therapeutic indices (tumor-to-normal tissue mean absorbed dose ratios) with monovalent [^177^Lu]Lu-ABD and bivalent [^177^Lu]Lu-Gemini radiohaptens in nude mice bearing orthotopic SW1222-luc CRC liver metastasis xenografts.

	[^177^Lu]Lu-ABD (3.7 MBq , 0.4 nmol)				[^177^Lu]Lu-Gemini (3.7 MBq , 0.2 nmol)			
Pretargeting interval	24h		48h		24h		48h	
	Estimated mean absorbed dose (cGy/MBq)	Therapeutic index	Estimated mean absorbed dose (cGy/MBq)	Therapeutic index	Estimated mean absorbed dose (cGy/MBq)	Therapeutic index	Estimated mean absorbed dose (cGy/MBq)	Therapeutic index
**Tumor**	32.88	/	11.82	/	125.43	/	119.88	/
**Blood**	2.14	15	1.54	8	3.50	36	2.40	50
**Heart**	1.39	24	0.65	18	3.76	33	3.58	34
**Lungs**	2.44	13	1.00	12	5.53	23	8.87	14
**Normal liver**	3.14	10	1.36	9	7.68	16	5.85	20
**Spleen**	2.28	14	1.04	11	6.24	20	5.31	23
**Stomach**	0.49	67	0.28	42	2.00	63	0.81	147
**Small intestine**	0.74	45	1.08	11	2.16	58	1.36	88
**Large intestine**	2.14	15	1.33	9	4.20	30	1.72	70
**Kidneys**	6.94	5	6.80	2	13.52	9	13.23	9
**Muscle**	0.49	68	0.19	62	1.00	125	0.70	171
**Bone**	2.42	14	0.22	53	1.61	78	0.84	143
**Ovaries**	2.38	14	1.03	11	6.83	18	3.66	33
**Uterus**	4.05	8	1.78	7	10.27	12	6.21	19

**Table 2:** GPA33 immunohistochemistry (IHC) signal positivity in recurrent hepatic tumors and extrahepatic metastases in nude mice (M1-M5) treated with three-step DOTA-PRIT with monovalent [^177^Lu]Lu-ABD (Group 1 (G1), 3 therapy cycles, 1 week apart) and bivalent [^177^Lu]Lu-Gemini (Group 2 (G2), single therapy cycle) (experimental protocol in Figure 5A).

Mouse #	Organs	IHC Signal Positivity	Survival (days)	Treatment
**G1M1**	Recurrent liver mass	> 90%	64	[^177^Lu]Lu-ABD
	Small intestine	70%		
	Axillary lymph node	> 90%		
	Diaphragm	> 90%		
**G1M2**	Recurrent liver mass	5%		
**G2M1**	Recurrent liver mass	70%	81	[^177^Lu]Lu-Gemini
**G2M2**	Recurrent liver mass	> 90%	113	
**G2M3**	Recurrent liver mass	> 90%		
	Pancreas	20%		
**G2M4**	Liver tumor scar tissue	NA	81	
**G2M5**	Liver minimal residual viable tumor cells	> 90%	68	
	Spinal cord	> 90%		
	Mesometrium minimal viable tumor cells	> 90%		
**G3M3**	Liver mass in control mouse	> 90%	11	no BsAb/[^177^Lu]Lu-ABD

## Abbreviations

ABD: aminobenzyl-DOTA
AUC: area under the curve
BLI: bioluminescence imaging
BsAb: bispecific antibody
CRC: colorectal cancer
CT: computed tomography
DOTA: 1,4,7,10-tetraazacyclododecane-1,4,7,10-tetraacetic acid
FDR: false discovery rate
GPA33: glycoprotein A33
H&E: hematoxylin and eosin
IHC: immunohistochemistry
IgG: immunoglobulin G
LD50: median lethal dose
MEM: minimal essential medium
MIP: maximum intensity projection
MPR: multiplanar reconstruction
MSK: Memorial Sloan Kettering Cancer Center
OCT: optimal cutting temperature
PBS: phosphate-buffered saline
PET: positron emission tomography
PRIT: pretargeted radioimmunotherapy
QAR: quantitative autoradiography
ROI: region-of-interest
SADA: self-assembling-disassembling antibody platform
scFv: single-chain fragment variable
SD: standard deviation
SEM: standard error of the mean
SPECT: single-photon emission computed tomography
TI: therapeutic index
%ID/g: percent injected dose per gram
T:NT: tumor-to-normal tissue ratio
