# Supplementary material for: GPA33-pretargeted radioimmunotherapy with mono- and bivalent DOTA-based Lu-177-labeled radiohaptens in a mouse orthotopic liver xenograft model of metastatic human colorectal cancer
Source: Theranostics. 2025 May 25;15(13):6274–89. doi: 10.7150/thno.107209 (PMC12159840; doi:10.7150/thno.107209)
Supplement: Supplementary file 1 — Supplementary figures and tables. [file thnov15p6274s1.pdf]

SUPPLEMENTARY MATERIAL

Synthesis of DOTA-Bn-thiouredo-PEG_4-thiouredo-Bn-DOTA (ABD-PEG_4-ABD, aka Gemini)

The linker 1,14-Diamino PEG_4, (3,6,9,12-tetraoxatetradecane-1,14-diamine) was obtained from Sigma-Aldrich (St. Louis, MO, USA). High purity metal-free DOTA chelators aminobenzyl DOTA (B-200, p-NH_2-Bn-DOTA) and para-isothiocyanatobenzyl DOTA (B-205, p-SCN-Bn-DOTA) were purchased from Macrocyclics (Dallas, TX, USA) and were used without further purification. Reaction monitoring and follow-up purifications were executed by either thin-layer chromatography (TLC) or on an AutoPurification high-performance liquid chromatography (HPLC) system (Waters Corporation, Milford, MA, USA) using an analytical C-18 column (Xbridge, Waters, 2.5-micron, 4.6 × 50 mm) as well as a preparative C-18 column (Xbridge, Waters, 5.0-micron, 19 × 150 mm) in combination with simultaneous detection by mass spectrometry, diode array, and evaporative light scattering.

1,14-Diamino PEG_4 (36 mg, 0.152 mmol) and DOTA-Bn-SCN.2.5 H_2O. 2.5 HCl (210 mg, 0.305 mmol) were dissolved in DMF/H_2O (1/1.5 mL respectively), treated with triethylamine Et_3N (0.15 mL), and the resulting mixture was stirred at room temperature overnight. Solvents were then removed by evaporation under reduced pressure, and the residue was purified by HPLC using the gradient 10–40% acetonitrile (containing 0.05% trifluoroacetic acid (TFA)) in water (containing 0.05% TFA). Lyophilization of product-containing fractions provided (160 mg, 78%) DOTA-Bn-thiourea-PEG_4-thiourea-Bn-DOTA (also known as (3,6,9,12-tetraoxatetradecane-1,14-diyl)bis(DOTA-benzyl thiourea) as a white foam.

General radiochemistry

Radiochemistry was performed in an appropriately shielded chemical fume hood equipped with electronic flow monitoring and sliding leaded glass windows. The activity of samples was measured with a CRC-55tR dose calibrator (Capintec Inc., Florham Park, NJ, USA) using manufacturer-recommended calibration settings. Buffers used for radiochemical synthesis were treated with 5% (w/v) Chelex ion exchange resin (BT Chelex 100 Resin, Bio-Rad Inc., Hercules, CA, USA) to remove adventitious heavy metals and 0.22-μm sterile filtered. Plasticware (such as pipette tips and microcentrifuge tubes) was sterile, trace metal grade/RNA grade. Radio-HPLC was performed on a Shimadzu Prominence HPLC system (Shimadzu Scientific Instruments, Somerset, NJ, USA) comprised of an LC-20AB dual pump module, DGU-20A3R degasser, SIL-20ACHT autosampler, SPD-20A UV-Vis detector, and an in-line Flow-Count B-FC-1000 PMT/NaI radioactivity detector (Bioscan/Eckert & Ziegler, Wilmington, MA, USA). The HPLC conditions were as follows: column: 4.6 × 250 mm Gemini-NX C18, 5 um, 100 Å (Phenomenex Inc., Torrance, CA, USA); mobile phase: A – 10 mM pH 5 NH_4OAc, and B – CH_3CN; flow rate: 1.0 mL/min; λ = 254 nm; injection volume: 5–20 μL; and gradient: 0% B to 40% B over 10 min. Samples of free radiometals, reaction mixtures, and purified products were diluted 1:5 in 5 mM DTPA or EDTA prior to analysis. Reaction progress was also monitored by radio-instant TLC (iTLC) using Agilent iTLC-SG strips eluted in 1) 50mM EDTA or 2) 1:1 MeOH/50mM NH_4OAc pH 5.5 and quantitated on a Bioscan AR-2000 (Bioscan/Eckert & Ziegler, Wilmington, MA, USA).

Radiolabeling of [^{177}Lu]Lu-ABD and [^{177}Lu]Lu-Gemini

Radiometal chelation of aminobenzyl-DOTA (ABD, a.k.a. DOTA-Bn, p-NH_2-Bn-DOTA) and Gemini (DOTA-Bn-thiouredo-PEG_4-thiouredo-Bn-DOTA, ABD-PEG_4-ABD) were performed as previously described with minor modifications [1-4]. Stock solutions of radiochemical precursors (unchelated ABD or Gemini) were made as 1 or 10 mM solutions in metal free water (Ultra Trace Water W9, Thermo Fisher Scientific, Waltham, MA, USA), aliquoted in PCR-grade, 0.5-mL microfuge tubes and stored at −20°C until use. Lutetium-177 isotope ([^{177}Lu]$LuCl_3$) was obtained from Isotopia Molecular Imaging (Petah Tikva, Israel) as a solution in 0.04-M HCl, with no carrier added and a specific molar activity of ~3561 GBq/mg (623 MBq/nmol, 16.8 mCi/nmol). A 100-mM stock solution of non-radioactive lutetium ($^{nat}LuCl_3$) was made by dissolving lutetium(III) chloride hexahydrate (Strem/Ascensus, Newburyport, MA, USA) in 0.1 M HCl (Thermo Fisher Scientific A508 diluted in metal free water). Reaction progress and radiochemical purity were evaluated by radio-HPLC and/or iTLC. Radiochemical products were diluted as needed in normal sterile saline (0.9% sodium chloride injection, USP, Hospira, Lake Forest, IL, USA), treated with 25 μM DTPA as a scavenger chelator, sub-micron sterile filtered (Millex 0.22 μM PES syringe filter, MilliporeSigma, Cork, Ireland), and used within several hours of manufacture. Once diluted in saline at the required injected activity concentration, radiolysis was not observed short-term (hours), and so radioprotectants were

generally not used. However, for longer than 4 h of storage, ^{177}Lu-labeled compounds were formulated in 50 μM ascorbate in saline and could be stored at 4°C for 24–48 hours without significant (<5%) degradation.

A typical [^{177}Lu]Lu-ABD radiolabeling with a molar activity (A_M) of 111 MBq/nmol (3 mCi/nmol) is as follows: ABD (p-NH_2-Bn-DOTA, 10 μL of 1 mM stock, 10 nmol) obtained from Macrocyclics (Dallas, TX, USA) was radiolabeled with 1.11 GBq (30 mCi) of [^{177}Lu]$LuCl_3$ in 100 μL of 1.0 M NaOAc (pH 5.3) buffer at 90 °C for 30 min. The reaction was briefly cooled, and quantitative incorporation of ^{177}Lu was confirmed by confirming an absence of free ^{177}Lu (DTPA) using radioHPLC. In a second incubation for 15 min at 90 °C, unchelated DOTA was backfilled by adding 10-fold excess nonradioactive lutetium (1 μL of 100 mM stock in 0.1 M HCl, 100 nmol). This short incubation with a moderate excess of ^{nat}Lu does not displace ^{177}Lu from the in-cage DOTA complex. Radiometal incorporation was nearly quantitative and purity was ≥ 99.5% by radioHPLC (t_R = 6.12 min (minor), 6.56 min (major), [^{177}Lu]Lu-DTPA was below detection at t_R ~2.5 min, **Figure S1A**). After cooling, the crude product was treated with 25 μM DTPA (~10-fold excess) to scavenge traces of free ^{177}Lu. After dilution in saline and sterile filtration, a typical isolated yield for a 1.11-GBq (30-mCi) run was 1.0–1.05 GBq (27–28 mCi) (> 90%), which includes losses due to sampling, transfers, and sterile filtration. In vivo experiments required tight control of injected activity and mass; for this example, since [^{177}Lu]Lu-ABD was labeled at A_M = 111 MBq/nmol and formulated in saline at 293 MBq/mL, each 150-μL mouse injection contained 0.4 nmol and 44 MBq (1.2 mCi). Two characteristic peaks are observed in the radiochromatogram of [^{177}Lu]Lu-ABD (**Figure S1A**) consistent with helical isomerism of the [M]DOTA complex [5]; these isomers do not readily interconvert, and both bind tightly to the anti-DOTA antibody scFv. As reported previously, unless the metal center is backfilled with nonradioactive metal, only the radiometallated ABD is recognized by the anti-DOTA scFv C825, which raises the effective molar activity of [^{177}Lu]Lu-ABD considerably in vivo and is thus governed by the specific activity of the radiometal as reported previously [1-3].
[^{177}Lu]Lu-ABD radiolabeling for the ex vivo biodistribution study was performed using the same protocol, apart from a lower molar activity (A_M) of 9.15 MBq/nmol (0.25 mCi/nmol), to obtain a final 150-μL mouse injection containing 0.4 nmol and 3.7 MBq (0.1 mCi) of [^{177}Lu]Lu-ABD.

A typical [^{177}Lu]LuGemini ([^{177}Lu]Lu-ABD-PEG_4-Lu-ABD) radiolabeling with a molar activity A_M = 222 MBq/nmol (6 mCi/nmol), in the same manner as [^{177}Lu]Lu-ABD radiolabeling, is as follows: [^{177}Lu]$LuCl_3$ (1.11 GBq, 30 mCi) was added to the Gemini precursor (5 nmol, 5 μL, 1 mM, DOTA-Bn-thiouredo-PEG_4-thiouredo-Bn-DOTA, ABD-PEG_4-ABD) in 100 μL of 1.0M NaOAc, pH 5.3, and heated to 90 °C for 30 min. To this reaction was added $^{nat}LuCl_3$ (1 μL, 100 nmol) and heated again (90 °C, 15 min) to backfill empty DOTA sites, cooled, sampled for radioHPLC (**Figure S1B**), and then quenched with DTPA after complete conversion was confirmed (no free ^{177}Lu by radioHPLC). Gemini efficiently chelated ^{177}Lu and no displacement of radiometal was observed during backfilling (radioHPLC purity of 98%, sum of all product/DOTA helical isomeric peaks (main peak t_R = 8.36 min)). For in vivo studies, this was followed by formulation in sterile saline, and 0.22 μm sterile filtration in the same manner for [^{177}Lu]Lu-ABD radiolabeling. The typical isolated yield for a 1.11-GBq (30 mCi) ^{177}Lu-Gemini run was 0.98–1.03 GBq (26.5–27.8 mCi) (>88%), which includes losses due to sampling, transfers, and sterile filtration. Since [^{177}Lu]Lu-Gemini was labeled at A_M = 222 MBq/nmol and formulated in saline at 293 MBq/mL, each 150-μL mouse injection contained 0.2 nmol and 44 MBq (1.2 mCi).
[^{177}Lu]Lu-Gemini radiolabeling for the ex vivo biodistribution study was performed using the same protocol, apart from a lower molar activity (A_M) of 18.3 MBq/nmol (0.5 mCi/nmol), to obtain a final 150-μL mouse injection containing 0.2 nmol and 3.7 MBq (0.1 mCi) of [^{177}Lu]Lu-Gemini.

MicroCT

During the 3-step DOTA-PRIT study, progression of the liver tumors was assessed by performing serial microCT scans using the Inveon PET/CT system (Siemens USA, Chicago, IL, USA). Long-lasting microCT contrast agent (ExiTron™ nano 12000, Miltenyi Biotec, Bergisch Gladbach, Germany) was administered intravenously in the mice tail vein (100 μL, immediately after the BsAb injection) for enhanced liver tumor visualization. This contrast medium, which avoids renal clearance due to its large size (> 6 nm), is eventually cleared from the bloodstream by phagocytic cells of the reticuloendothelial system [6], primarily in the normal liver and spleen, and a single injection is sufficient for enhanced visualization of the hepatic tumor over the entire course of the therapy study. The mice were anesthetized with 1.5–2% isoflurane and placed in the scanner in a prone position. A total of 360 CT projections were acquired over a 180° rotation with the

following parameters: a voltage of 70 kVp, current of 500 µA, and an exposure time of 275 ms per projection. The images were reconstructed using a modified Feldkamp cone beam reconstruction algorithm to generate 512 × 512 × 768 voxel image volumes (0.197 × 0.197 × 0.197 mm voxel dimensions). Post-reconstruction, Vivoquant software (Invicro, Boston, MA, USA) was used to analyze the CT images and assess liver tumor size.

SPECT

All SPECT scans were obtained using a 4-head γ-camera dedicated small-animal scanner (NanoSPECT/CT, Mediso Medical Imaging Systems, Budapest, Hungary) with an animal palette heated to 37°C. High spatial resolution pinhole collimators (NS108) with an energy range optimized for the 113 and 208 keV ^{177}Lu γ-rays (6.4 and 11% abundance, respectively) were used. After anesthesia of the mice with 1.5%–2% isoflurane, a 360° small-animal SPECT was acquired with a 60-s projection image duration. Bioscan HiSPECT software (HiSPECT. Bioscan Inc., Washington, DC, USA) was used for iterative image reconstruction and fusion of CT and SPECT images. Post-reconstruction, Vivoquant software (Invicro, Boston, MA, USA) was used to analyze the SPECT images.

Bioluminescent imaging

Bioluminescent imaging (BLI) using the IVIS Spectrum In Vivo Imaging System (PerkinElmer, Inc., Waltham, MA, USA) was utilized to monitor tumor growth every 7–15 days following first-cycle radiohapten injection. Images were obtained 10 min after intraperitoneal injection of 200-µL D-luciferin (15 mg/mL in PBS, Gold Biotechnology, St. Louis, MO, USA), with exposure times ranging from 1–30 s. The mice were anesthetized with 1.5–2% isoflurane and placed in the supine position during image acquisition. The Aura Image Analysis Software v.4.0 (Spectral Instruments Imaging, LLC, Tucson, AZ, USA) was used to analyze the BLI images. The total photon flux (photons/s) was calculated by drawing regions of interest (ROIs) encompassing the entire abdominal cavity, from the axilla down to the base of the tail. The same ROIs were restored between each BLI timepoint for measurement consistency.

Histopathology

All tissues were collected and fixed in 10% neutral buffered formalin (NFB) for histopathology. After at least 72 h of fixation in 10% NFB, samples were trimmed and processed in ethanol and xylene and embedded in paraffin in a Leica ASP6025 tissue processor (Leica, Deer Park, IL, USA). Bones were decalcified in a solution of formic acid and formaldehyde after fixation and prior to processing. Paraffin blocks were sectioned at 5-µm thickness and stained with hematoxylin and eosin (H&E). All tissues were examined by a board-certified veterinary pathologist (S.M.). A complete necropsy included macroscopic and microscopic evaluation of the following organs: heart, lungs, thymus, kidneys, liver, gallbladder, stomach, duodenum, jejunum, ileum, cecum, colon, mesenteric lymph node, salivary glands, submandibular lymph node, uterus, cervix, vagina, urinary bladder, spleen, pancreas, adrenals, ovaries, oviducts, trachea, esophagus, thyroid, parathyroid, skin (trunk, perigenital, head), mammary glands, bones (femur, tibia, sternum, vertebrae, skull), bone marrow (femur, tibia, sternum, vertebrae), stifle joint, skeletal muscles (hind limb, spine), nerves (hind limb, spine), spinal cord, oral cavity, teeth, nasal cavity, eyes, harderian gland, pituitary, brain, and ears. Stained slides were digitalized (bright field × 20) on the Slideview VS200 research slide scanner (Olympus Corporation, Tokyo, Japan) and visualized using Olympus Net Image Server SQL.

Immunohistochemistry

Immunohistochemical staining for GPA33 was performed on formalin-fixed paraffin-embedded samples, using an in-house purified mouse antibody clone 175-285-30 provided by the Memorial Sloan Kettering Cancer Center Ludwig Institute for Cancer Research, at a 1:20 dilution (5 µg/mL). Further information about the antibody can be found in a previous publication [7]. Staining was performed on the Leica Bond-3 auto staining system (Leica, Deer Park, IL, USA), using heat-based antigen retrieval, a citrate-based buffer solution (AR9961; Leica, Bond Epitope Retrieval Solution 1, 30 min), 30-min primary incubation time, and a polymer detection system (DS9800; Leica, Bond Polymer Refine Detection).

Hematology

Blood samples (75-100 µL) were collected from the retroorbital sinus or submandibular facial vein and collected in ethylenediaminetetraacetic acid (EDTA) tubes (Microtainer, BD, Franklin Lakes, NJ, USA, average of 1 mg EDTA per tube). Complete blood counts (CBC) with differentials were performed using an

automated hematology analyzer (Element HT5, Heska, Loveland, CO, USA). The following parameters were obtained for analysis: white blood cell count (WBC), neutrophils absolute count and percentage (NEU), lymphocytes absolute count and percentage (LYM), monocytes absolute count and percentage (MONO), eosinophils absolute count and percentage (EOS), basophils absolute count and percentage (BAS), red blood cell count (RBC), hemoglobin (HGB), hematocrit (HCT), mean corpuscular volume (MCV), mean corpuscular hemoglobin (MCH), mean corpuscular hemoglobin concentration (MCHC), red cell distribution width percentage (RDW %), platelet count (PLT), and mean platelet volume (MPV). Blood samples from 5 experimentally naïve immunodeficient mice were used as reference, by calculating the mean ± 2 SD.

Biodistribution assay

Mice were humanely euthanized by CO_2 asphyxiation and tissues of interest were harvested immediately after. Activity concentrations were determined for each tissue sample by counting them in a γ-counter (Hidex Automatic Gamma Counter, Hidex Oy, Turku, Finland) set to a 155–425 KeV energy window (^{177}Lu). Count rates were background- and decay-corrected, converted to activities using a system calibration factor specific for the isotope, normalized to the administered activity, and expressed as percent injected dose per gram (%ID/g).

Quantitative autoradiography (QAR)

SW1222-luc tumor-bearing mice received an intravenous injection of anti-GPA33 BsAb (250 μg/1.19 nmol), at t = −24 h for the monovalent radiohapten [^{177}Lu]Lu-ABD mice, and at t = −48h for the bivalent radiohapten [^{177}Lu]Lu-Gemini mice (**Figure 1**). A CCA16-DOTA.Y dendrimer clearing agent (CA) was then injected intravenously (25 μg/2.8 nmol), at t= −4 h. The monovalent radiohapten mice received 44.4 MBq (0.4 nmol) of [^{177}Lu]Lu-ABD at t = 0 h, and the bivalent radiohapten mice 44.4 MBq (0.2 nmol) of [^{177}Lu]Lu-Gemini at t = 0 h. Immediately after euthanasia of the mice, 5 days post radiohapten injection, the SW1222-luc liver tumors were resected and part of the tumor along with some adjacent normal liver parenchyma were trimmed for QAR. Each sample was placed into a cryomold, which was then filled with optimal cutting temperature (OCT) compound (Sakura Finetek USA, Inc., Torrance, CA, USA), and snap frozen by partially immersing the cryomold in a bath of dry ice-cooled isopentane. Once frozen, the cryomold was stored at −80°C until ready for sectioning. A series of sequential sections of 7-μm thickness were cut using a Leica CM 1860 Cryostat (Leica, Deer Park, IL, USA) and placed in a film cassette against a phosphor imaging plate (Fujifilm BAS-MS2325; Fuji Photo Film, Valhalla, NY, USA) for a 24-h exposure period at −20 °C. The phosphor imaging plate was then read at a pixel resolution of 25 μm with a Typhoon 7000 IP plate reader (GE Healthcare, Chicago, IL). The total activity of each histological section was measured by counting the whole histological slide in a γ-counter (Hidex Automatic Gamma Counter, Hidex Oy, Turku, Finland) set to a 155−425 KeV energy window (^{177}Lu).

Image analysis was performed using ImageJ 1.54f software. ROIs were drawn digitally around the whole samples and respective tumor regions and background corrected values were converted into %ID/g relative activities using the previously counted total activity of each section. After ^{177}Lu decay, the OCT-frozen tumor slides used for QAR were stained with H&E for tumor morphology assessment.

Dosimetry

Serial biodistribution data were used to calculate dosimetry. Isotope decay-adjusted activity was integrated over time, with the conservative assumption that all β-emissions were locally absorbed and that there was no biological clearance after the last timepoint (120 h), with the decay corrected tissue %ID/g remaining constant thereafter. The ^{177}Lu equilibrium dose constant for non-penetrating radiations (8.49 g-cGy/MBq-h) was used to estimate the tumor-to-tumor and select organ-to-organ self-mean absorbed doses (in cGy/MBq), assuming complete local absorption of the ^{177}Lu β-rays only and ignoring the γ-ray and non-self-dose contributions. To calculate the blood time integrated activity, the blood time activity curve was modeled as a single exponential from 0 to 2 h. Trapezoid integration was then used from 2 to 120 h and physical decay only after 120 h.

A

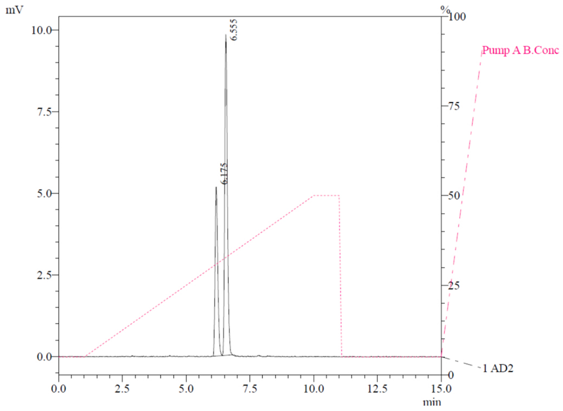

Peak #	Retention Time (min)	Area	Height	Area %	Height %
1	6.175	37768	5171	34.755	34.475
2	6.555	70901	9828	65.245	65.525
**Total**		108669	14999	100.000	100.000

B

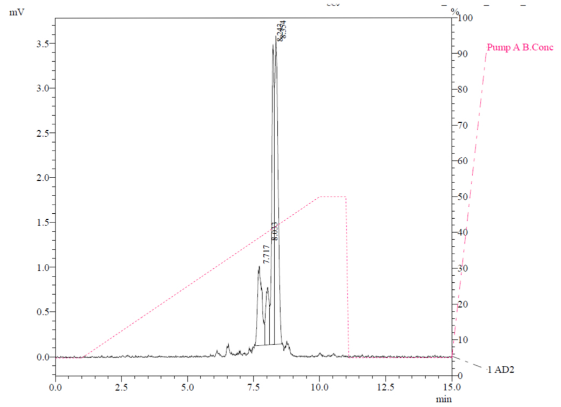

Peak #	Retention Time (min)	Area	Height	Area %	Height %
1	7.717	10556	886	15.658	10.626
2	8.033	5717	643	8.481	7.720
3	8.243	22871	3357	33.926	40.275
4	8.354	28270	3449	41.935	41.379
**Total**		67414	8335	100.000	100.000

Figure S1. Radio-HPLC chromatograms of (A) [^{177}Lu]Lu-ABD and (B) [^{177}Lu]Lu-Gemini.

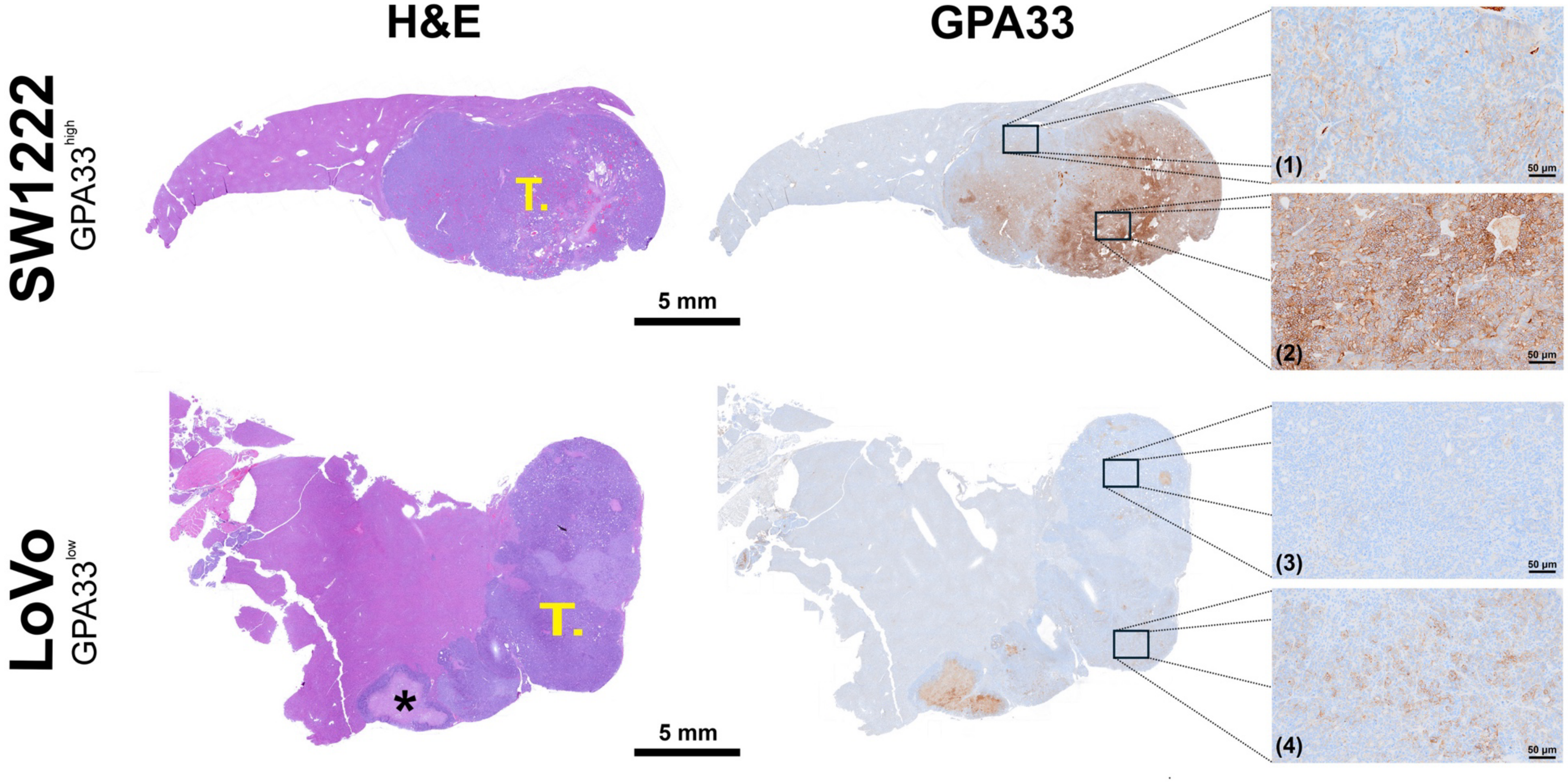

Figure S2. Differential GPA33 expression between SW1222-luc (GPA33^{high}) and LoVo (GPA^{low}) untreated liver tumors. Representative H&E micrograph and GPA33 immunostaining of GPA33^{high} SW1222-luc and GPA33^{low} LoVo liver tumors. On the SW1222-luc tumor section, mildly heterogeneous GPA33 immunostaining is noted, with a gradient from weak staining (upper left whole tumor image and zoomed image (1)) to strong positivity (bottom right of the whole tumor image and zoomed image (2)), with overall ~80% of tumor cells showing GPA33 positivity. In comparison, there is absent (3) to low, scattered (4) GPA33 immunostaining in the LoVo tumor (~5% IHC signal positivity). The asterisk marks a necrotic area, showing non-specific staining on IHC. T.: tumor.

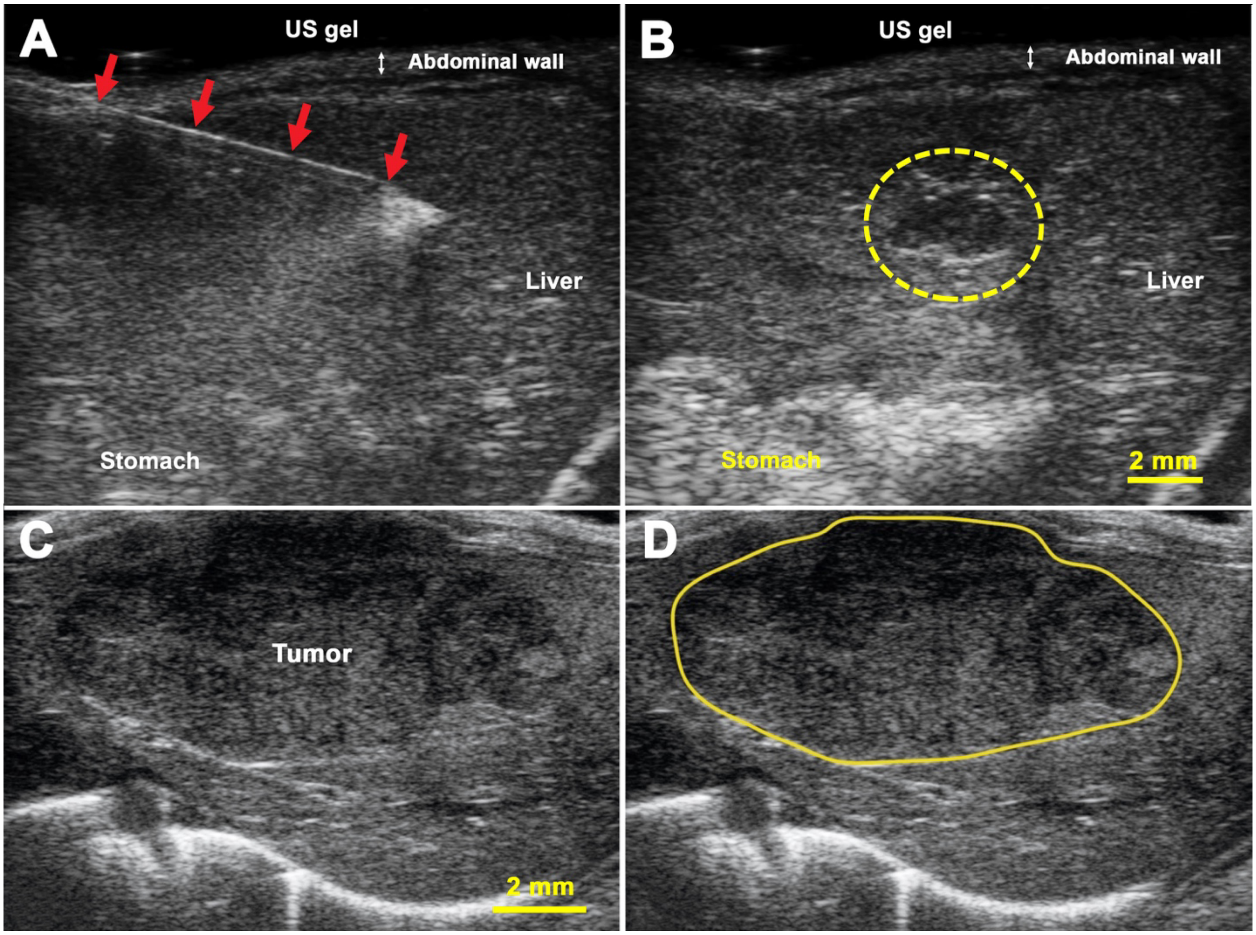

Figure S3 and Supplementary Video 1. Ultrasound-guided intraparenchymal liver inoculation of human colorectal cancer in mice. A. Percutaneous ultrasound-guided needle insertion (red arrows) in the liver. B. Small intraparenchymal liver cavitation post cell suspension inoculation (20 μL, 1 million SW1222-luc cells). C. B-mode transverse ultrasound image of the liver of a mouse 3 weeks post inoculation. A well-defined oval hypoechoic intraparenchymal colorectal cancer liver metastasis xenograft is present. D. The colorectal cancer liver metastasis is delineated by a continuous yellow line.

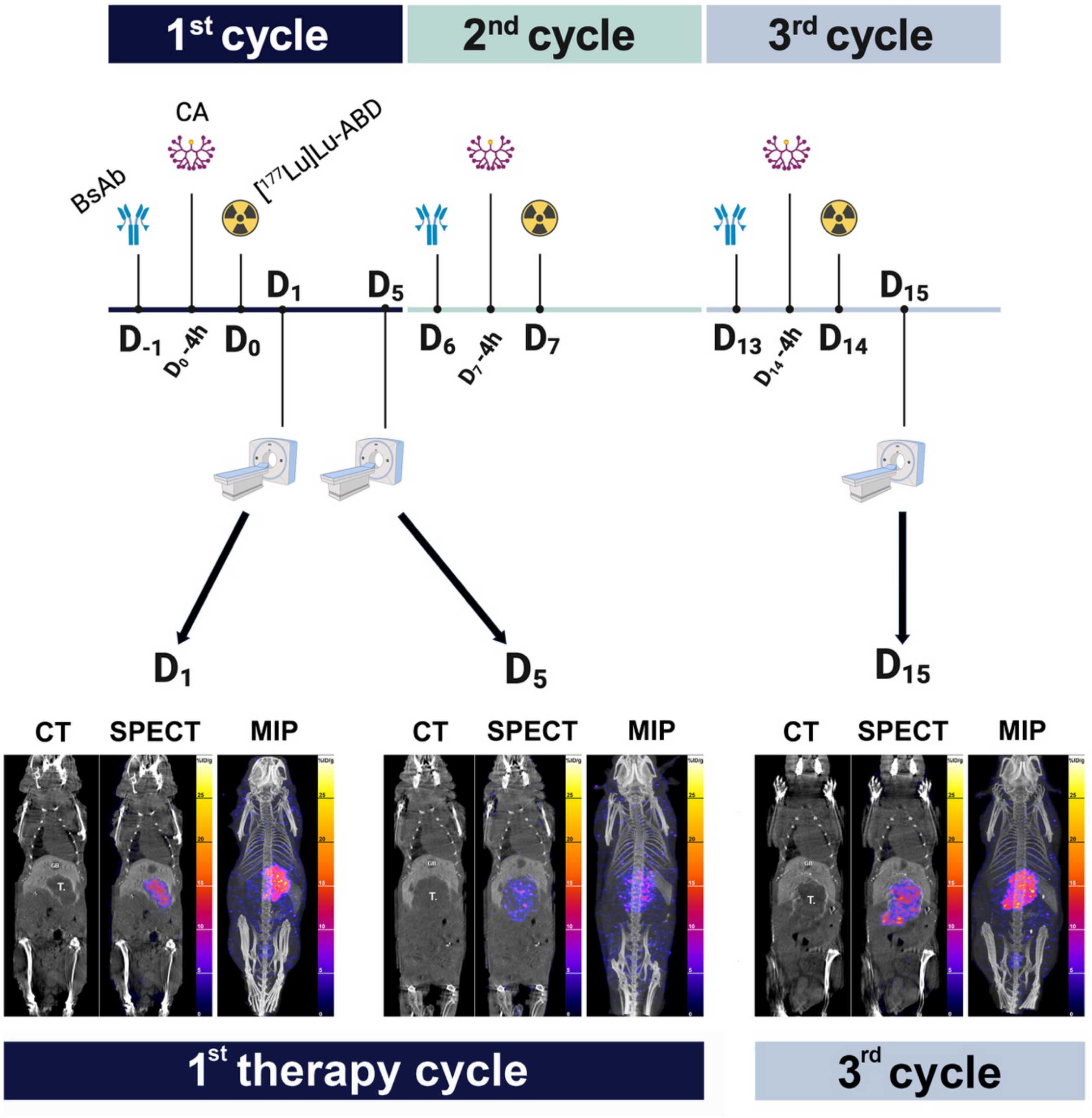

Figure S4. Retargeting in multicycle [^{177}Lu]Lu-ABD 3-step DOTA-PRIT. Coronal multiplanar reconstruction (MPR) and maximum intensity projection (MIP) SPECT/CT images acquired 24 h (D_1) and 120 h (D_5) after [^{177}Lu]Lu-ABD injection in the 1st treatment cycle (left), and 24 h (D_{15}) after [^{177}Lu]Lu-ABD injection in the 3rd treatment cycle (right) in the same mouse. High tumor uptake is noted 24 h after [^{177}Lu]Lu-ABD injection in the 1st treatment cycle (D_1), with progressive tumor size and decreased tumoral uptake noted 120 h (D_5) after the injection. On the images acquired 24 h after [^{177}Lu]Lu-ABD injection in the 3rd cycle (D_{15}), high tumor retargeting is evident, with relatively unchanged tumor size overtime. T.: tumor; GB: gallbladder.

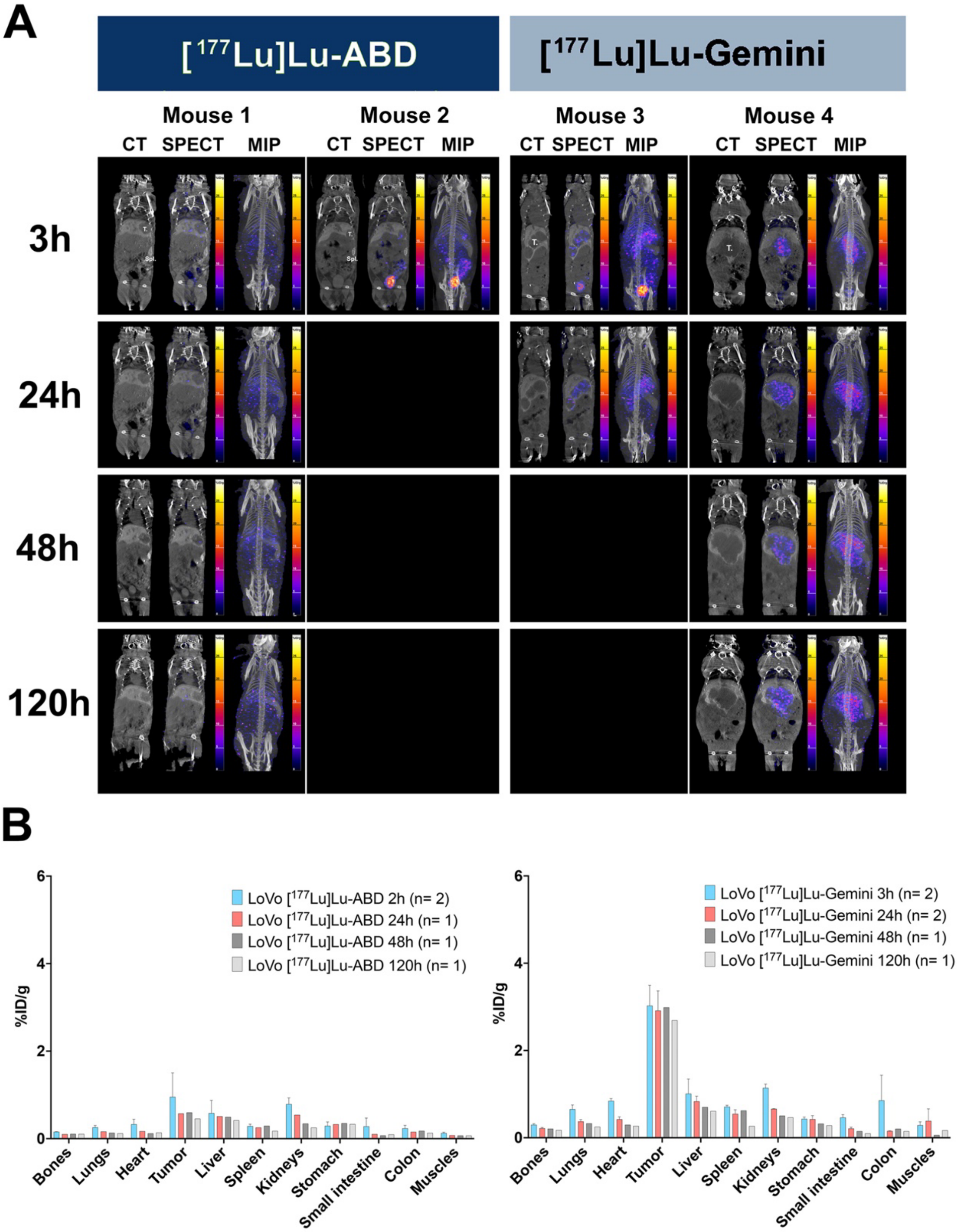

Figure S5. 3-step DOTA-PRIT SPECT/CT studies with [^{177}Lu]Lu-ABD and [^{177}Lu]Lu-Gemini in low GPA33-positive (LoVo) colorectal cancer liver metastasis tumor-bearing mice. A. Coronal multiplanar reconstruction (MPR) and maximum intensity projection (MIP) SPECT images acquired 3 h, 24 h, 48 h, and 120 h after 44.4 MBq injection of [^{177}Lu]Lu-ABD (0.4 nmol) and [^{177}Lu]Lu-Gemini (0.2 nmol). Superior tumor targeting and retention are noted with [^{177}Lu]Lu-Gemini, compared with [^{177}Lu]Lu-ABD which shows negligible tumor uptake. T.: hepatic tumor; Spl.: spleen. B. [^{177}Lu]Lu-ABD and [^{177}Lu]Lu-Gemini biodistribution based on SPECT. Values are represented as means, and error bars represent standard error of the mean (SEM).

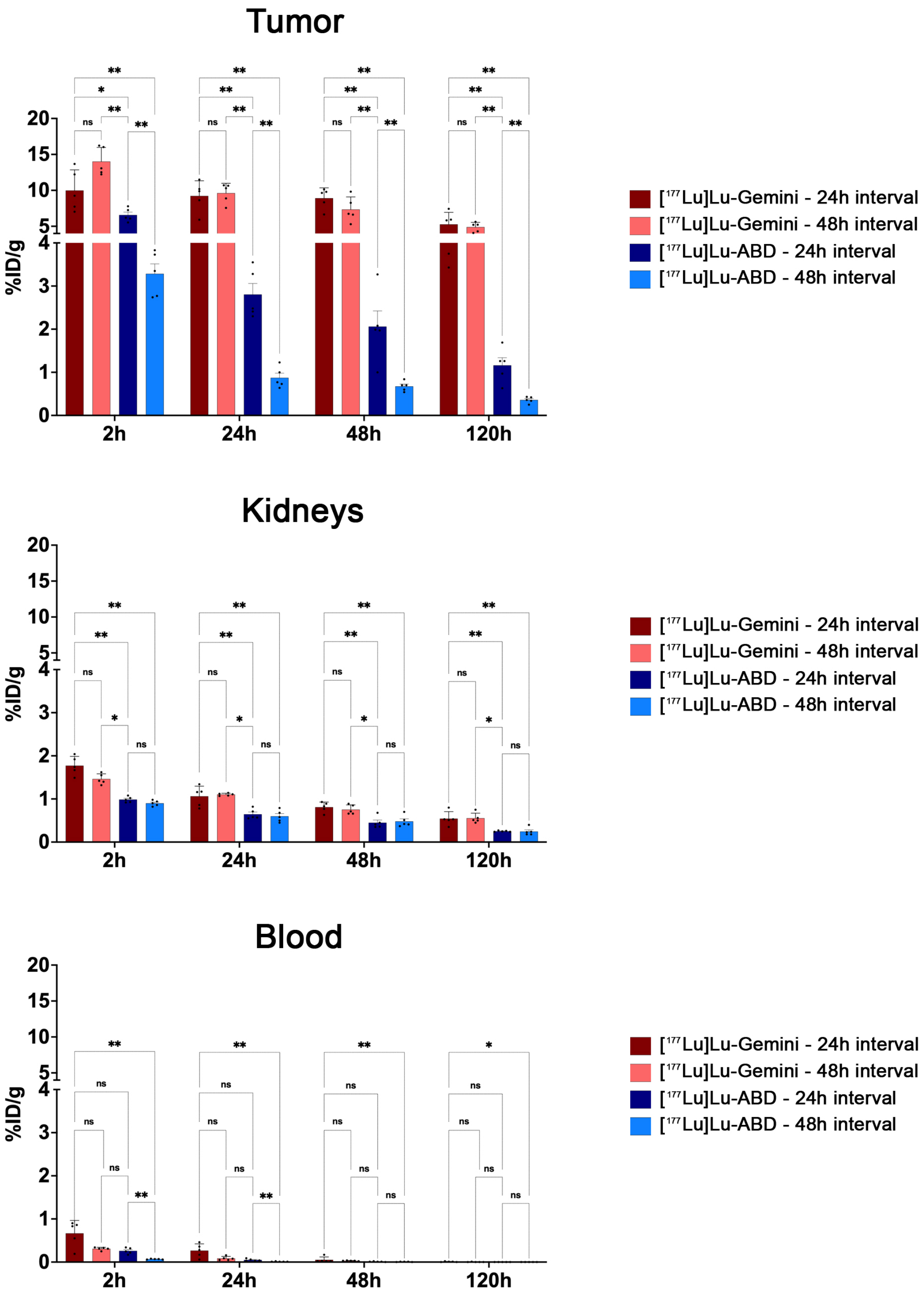

Figure S6. Tumor, kidneys, and blood ex vivo biodistribution after [^{177}Lu]Lu-ABD (3.7 MBq, 0.4 nmol) and [^{177}Lu]Lu-Gemini (3.7 MBq, 0.2 nmol) 3-step DOTA-PRIT. For each radiohapten, 2 different pretargeting intervals (24h and 48h) were evaluated between the BsAb and radiohapten injection. Statistical differences between groups at each time point were assessed using Mann-Whitney U tests. To account for multiple comparisons, p-values were adjusted using the two-stage step-up method of Benjamini, Krieger, and Yekutieli with a target false discovery rate (FDR) of 5%. Results were considered statistically significant when the FDR-adjusted p-value (q) was less than 0.05. ns = not significant; * FDR-adjusted p-value (q) ≤ 0.05; ** FDR-adjusted p-value (q) ≤ 0.01.

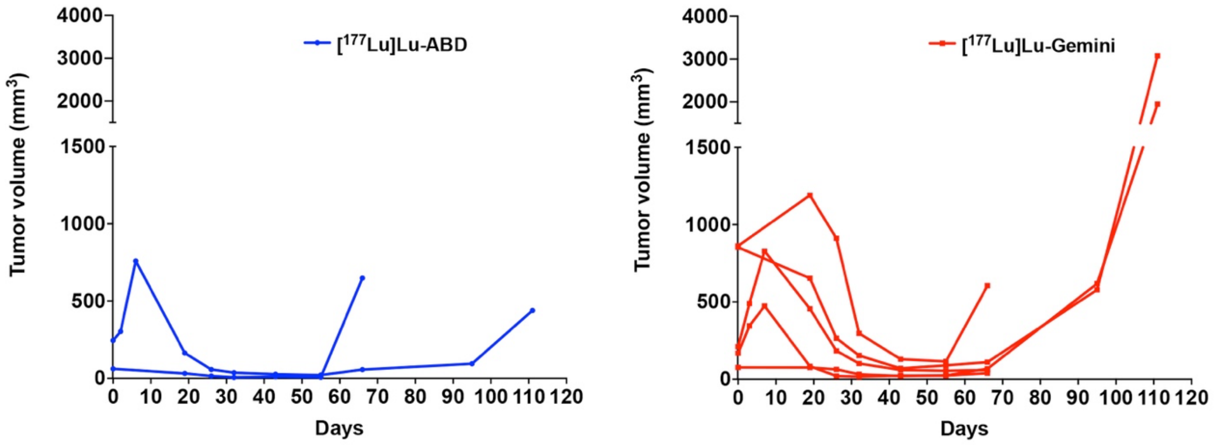

Figure S7. SW1222-luc tumor volumes from serial CT studies in nude mice after 3 cycles of 44.4 MBq [^{177}Lu]Lu-ABD (0.4 nmol) and a single cycle of 44.4 MBq [^{177}Lu]Lu-Gemini (0.2 nmol). Only 2/5 mice from the [^{177}Lu]Lu-ABD group could be imaged serially with CT due to perivenous extravasation of the positive contrast medium during intravenous injection in the other 3 mice.

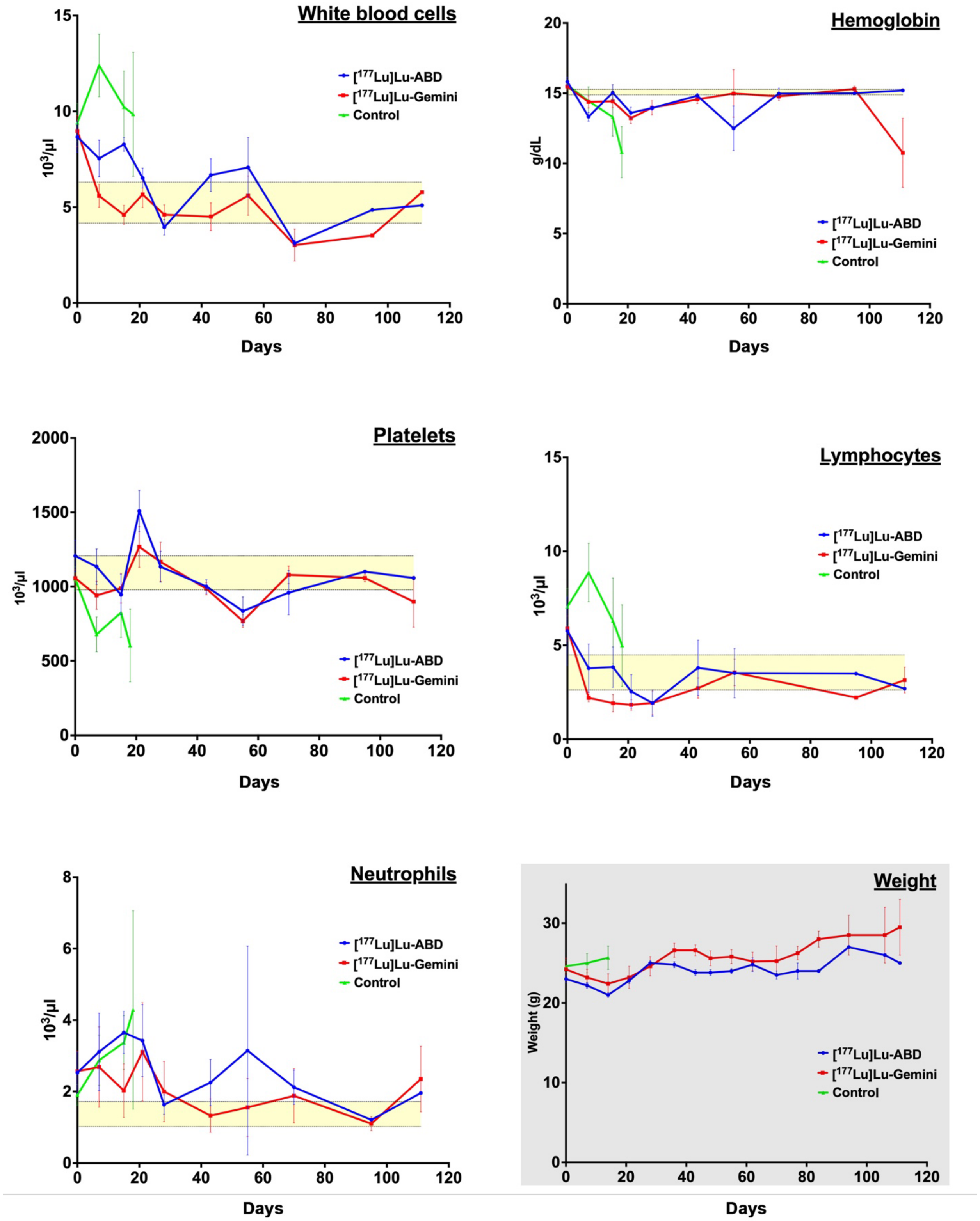

Figure S8. Serial complete blood count and weight results (mean ± standard error of the mean) in the 3-step DOTA-PRIT study. The yellow range represents 2 standard deviations of the mean of an untreated littermate group (n = 5) of nude female mice.

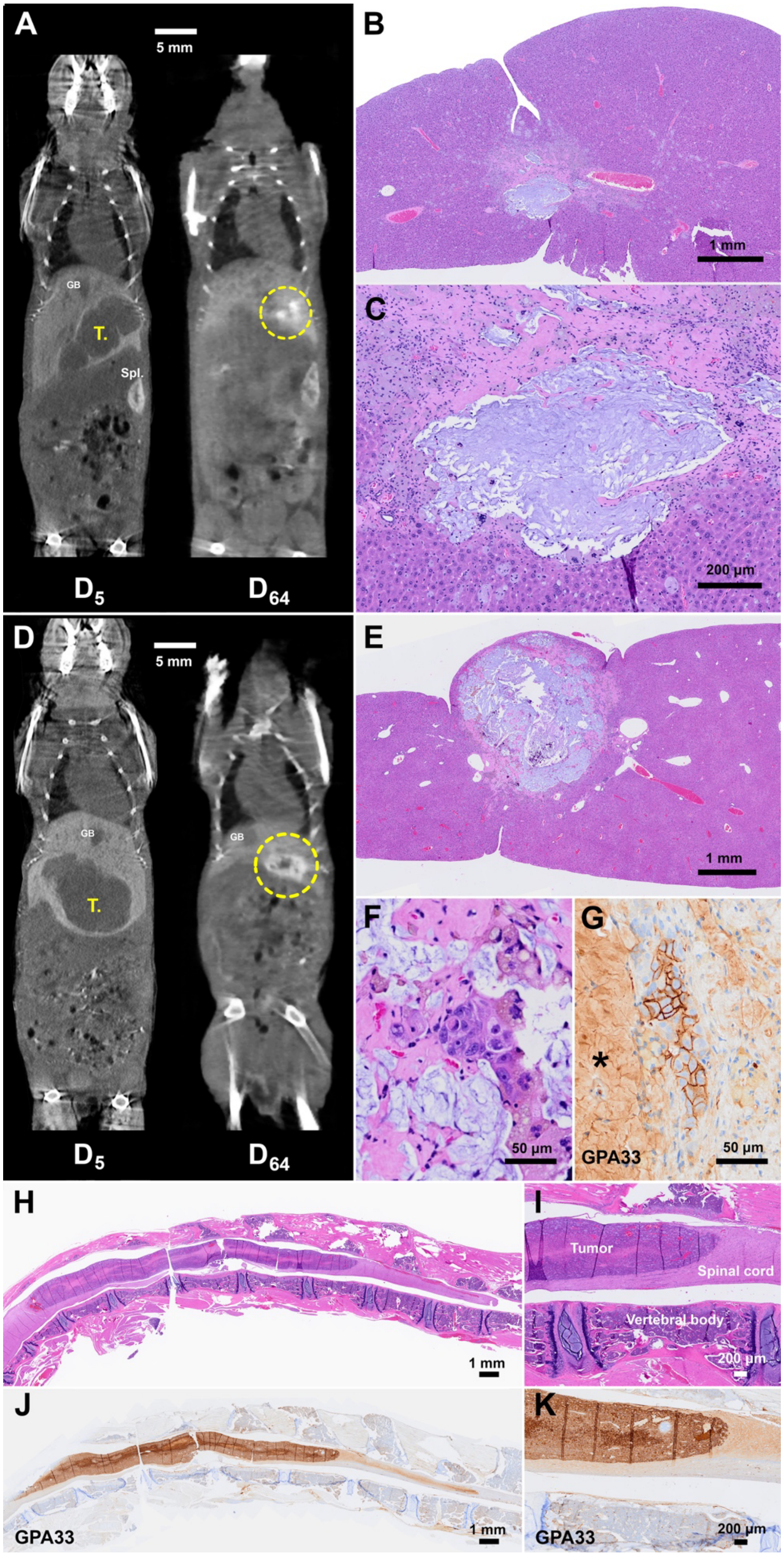
A
5 mm
GB
T.
Spl.
D5
D64
B
1 mm
C
200 µm
D
5 mm
GB
T.
GB
D5
D64
E
1 mm
F
50 µm
G
*
GPA33
50 µm
H
1 mm
I
Tumor
Spinal cord
Vertebral body
200 µm
J
GPA33
1 mm
K
GPA33
200 µm

Figure S9. Representative histology of two SW1222-luc colorectal cancer liver metastases that responded to 3-step DOTA-PRIT . A. Coronal multiplanar reconstruction (MPR) CT images of a mouse (G2M4 in Table 2) 5 (D_5) and 64 (D_{64}) days after single cycle of 44.4 MBq [^{177}Lu]Lu-Gemini (0.2 nmol) 3-step DOTA-PRIT. The tumor size decreased from 474 mm^3 to 39 mm^3, with a rim of contrast medium accumulating around the residual tumor (yellow dashed circle) on the D_{64} CT image. **B.** Low and **C.** high magnification hematoxylin and eosin (H&E) micrographs of the liver tumor site of the same mouse 81 days post treatment. Focal scar, composed of fibrosis, necrotic debris, mucin, and granulomatous inflammation is present at the tumor site, without evidence of viable tumor cells. GPA33 immunohistochemistry (IHC) (not shown) also confirmed the absence of viable tumor cells. **D.** Coronal MPR coronal images of a mouse (G2M5 in Table 2) 5 (D_5) and 64 (D_{64}) days after single cycle of 44.4 MBq [^{177}Lu]Lu-Gemini (0.2 nmol) 3-step DOTA-PRIT. The tumor is circled in yellow at D_{64}, and its size decreased from 829 mm^3 to 59 mm^3. **E.** Low and **F.** high magnification H&E micrographs of the liver tumor site of the same mouse 68 days post treatment. A small, poorly cellular tumor (adenocarcinoma), composed mostly of fibrosis, necrotic debris, mucin, and granulomatous inflammation, with few viable neoplastic cells is present post treatment**. G.** The residual tumor cells have a strong, membranous GPA33 expression (> 90% IHC signal positivity). A large necrotic area, which shows non-specific staining on IHC, is marked with an asterisk. **H.** Low and **I.** high magnification H&E micrographs of a large spinal metastasis in the same mouse (G2M5). A large highly cellular spinal cord metastasis is present, compressing the adjacent normal spinal cord. **J.** Low and **K.** high magnification GPA33 IHC micrographs show strong diffuse GPA33 expression (> 90% IHC signal positivity) of this metastatic spinal cord tumor. T.: tumor, GB: gallbladder, spl.: spleen.

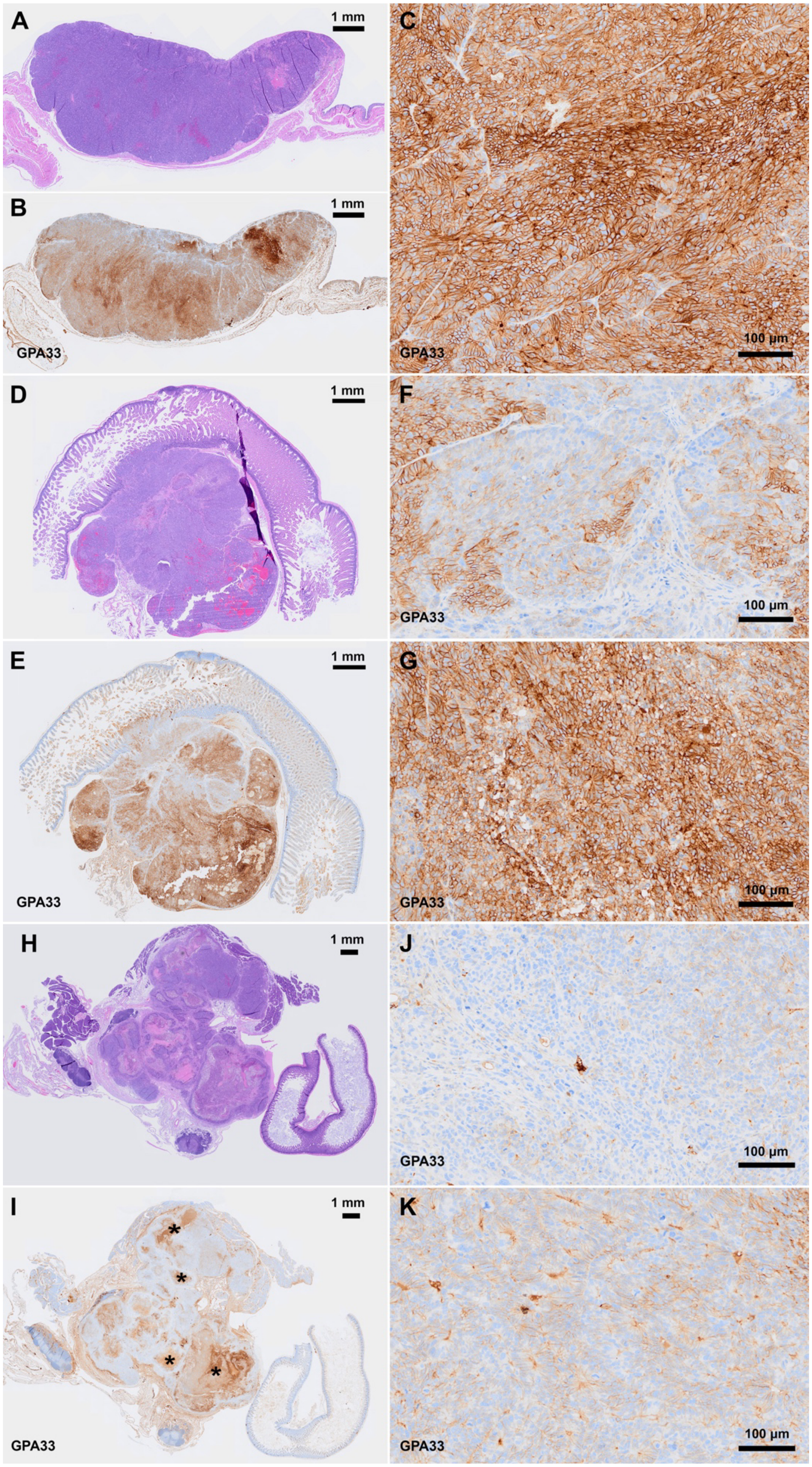
A
1 mm
B
1 mm
GPA33
C
GPA33
100 µm
D
1 mm
F
GPA33
100 µm
E
1 mm
GPA33
G
GPA33
100 µm
H
1 mm
J
GPA33
100 µm
I
1 mm
*
*
*
*
GPA33
K
GPA33
100 µm

Figure S10. GPA33 differential expression in metastatic SW1222-luc CRC tumors post 3-step DOTA-PRIT (experimental 3-step DOTA-PRIT protocol in Figure 5A). A.B.C. Large metastatic lesion in the diaphragm of a mouse (G1M1 in Table 2) 64 days after 3 treatment cycles of 44.4 MBq [^{177}Lu]Lu-ABD (0.4 nmol) DOTA-PRIT. The tumor has a strong, diffuse, membranous GPA33 expression, with 90% of the tumor cells strongly expressing GPA33. **D.E.F.G.** Large exophytic metastatic lesion in the small intestine of the same mouse as images A–C (G1M1 in Table 2). Most of the tumor cells (70% immunohistochemistry (IHC) signal positivity) have a strong, membranous GPA33 expression (image **G**), although patchy areas of GPA33(−) cells are noted in the periphery of the tumor (image **F**). **H.I.J.K.** Large pancreatic metastatic SW1222-luc CRC tumor in a mouse (G2M3 in Table 2) 113 days after a single therapy cycle of 44.4 MBq [^{177}Lu]Lu-Gemini (0.2 nmol) DOTA-PRIT. Only a small part of the tumor (20%) exhibits mild GPA33 positivity (image **K**), while the rest of the tumor has negligible GPA33 signal positivity (image **J**). Large necrotic areas, which show non-specific GPA33 staining on IHC, are marked with an asterisk.

Table S1. Ex vivo biodistribution results of [^{177}Lu]Lu-ABD (3.7 MBq, 0.4 nmol) and [^{177}Lu]Lu-Gemini (3.7 MBq, 0.2 nmol) 3-step DOTA-PRIT.

[^{177}Lu]Lu-ABD, 3.7 MBq, 0.4 nmol (%ID/g) – 24 h interval between antibody and clearing agent injection.

	**2 h**					**24 h**					**48 h**					**120 h**				
	M1	M2	M3	M4	M5	M1	M2	M3	M4	M5	M1	M2	M3	M4	M5	M1	M2	M3	M4	M5
Blood	0.34	0.17	0.25	0.22	0.32	0.09	0.03	0.06	0.03	0.04	0.01	0.02	0.01	0.01	0	0	0	0	0	0
Tumor	6.19	7.79	6.08	7.25	5.54	3.55	2.3	2.41	2.48	3.28	2.08	1.99	2.63	1.58	0.8	1.26	0.63	1.69	1.26	0.97
Heart	0.19	0.2	0.18	0.15	0.18	0.11	0.1	0.13	0.14	0.07	0.06	0.08	0.08	0.06	0.05	0.05	0.05	0.06	0.07	0.07
Lungs	0.6	0.37	0.41	0.42	0.43	0.22	0.29	0.24	0.27	0.14	0.33	0.12	0.09	0.21	0.13	0.06	0.06	0.1	0.07	0.09
Liver	0.33	0.46	0.74	0.46	0.3	0.21	0.16	0.23	0.17	0.19	0.14	0.15	0.2	0.23	0.22	0.12	0.09	0.17	0.16	0.11
Spleen	0.32	0.16	0.2	0.18	0.19	0.17	0.13	0.15	0.15	0.17	0.09	0.11	0.12	0.08	0.12	0.09	0.08	0.15	0.12	0.11
Stomach	0.06	0.11	0.08	0.06	0.08	0.02	0.02	0.03	0.03	0.05	0.03	0.02	0.05	0.02	0.02	0.02	0.02	0.02	0.01	0.03
Sm.intestine	0.28	0.29	0.25	0.26	0.23	0.06	0.03	0.07	0.05	0.05	0.03	0.04	0.05	0.03	0.03	0.03	0.02	0.02	0.01	0.02
Lg.intestine	1.58	1	1.03	1.23	2.42	0.05	0.07	0.08	0.06	0.09	0.04	0.08	0.06	0.06	0.18	0.02	0.02	0.03	0.02	0.02
Kidneys	1.08	0.98	0.93	0.92	1.02	0.58	0.71	0.82	0.57	0.55	0.36	0.35	0.45	0.41	0.68	0.26	0.24	0.25	0.24	0.27
Muscle	0.06	0.05	0.05	0.05	0.05	0.03	0.04	0.03	0.03	0.03	0.01	0.02	0.02	0.02	0.02	0.02	0.02	0.02	0.02	0.04
Tail	0.15	0.11	0.14	0.13	0.15	0.15	0.14	0.17	0.13	0.15	0.15	0.12	0.12	0.14	0.12	0.11	0.12	0.09	0.13	0.15
Bone	0.58	0.7	0.31	0.55	0.33	0.1	0.14	0.29	0.07	0.1	0.07	0.06	0.08	0.09	0.07	0.05	0.06	0.05	0.06	0.05
Ovaries	0.18	0.17	0.17	0.2	0.23	0.19	0.07	0.15	0.09	0.08	0.09	0.12	0.11	0.11	0.09	0.07	0.14	0.15	0.15	0.11
Uterus	0.33	0.28	0.34	0.23	0.45	0.15	0.17	0.19	0.21	0.15	0.15	0.14	0.1	0.1	0.1	0.18	0.16	0.18	0.37	0.25

[^{177}Lu]Lu-ABD, 3.7 MBq, 0.4 nmol (%ID/g) – 48 h interval between antibody and clearing agent injection.

	**2 h**					**24 h**					**48 h**					**120 h**				
	M1	M2	M3	M4	M5	M1	M2	M3	M4	M5	M1	M2	M3	M4	M5	M1	M2	M3	M4	M5
Blood	0.07	0.08	0.07	0.06	0.06	0.01	0.01	0.01	0.02	0.01	0	0.01	0	0.01	0.01	0	0	0	0	0
Tumor	3.73	3.83	2.77	2.74	3.35	0.77	0.64	0.73	1.23	1	0.54	0.7	0.7	0.84	0.6	0.34	0.36	0.42	0.43	0.25
Heart	0.08	0.08	0.07	0.07	0.06	0.04	0.03	0.03	0.05	0.04	0.03	0.04	0.03	0.04	0.03	0.03	0.03	0.03	0.04	0.02
Lungs	0.23	0.21	0.12	0.19	0.13	0.07	0.08	0.06	0.07	0.06	0.04	0.05	0.04	0.13	0.04	0.04	0.07	0.03	0.03	0.02
Liver	0.33	0.49	0.31	0.38	0.36	0.08	0.06	0.08	0.09	0.09	0.05	0.08	0.08	0.09	0.05	0.05	0.06	0.04	0.04	0.04
Spleen	0.09	0.1	0.1	0.15	0.09	0.06	0.06	0.06	0.06	0.07	0.04	0.06	0.05	0.09	0.05	0.06	0.04	0.04	0.06	0.04
Stomach	0.07	0.04	0.05	0.06	0.09	0.02	0.01	0.02	0.04	0.03	0.01	0.01	0.02	0.01	0.02	0.01	0.01	0.01	0.01	0.01
Sm.intestine	1.28	0.36	1.24	0.99	0.32	0.03	0.02	0.02	0.02	0.02	0.02	0.02	0.02	0.02	0.02	0.01	0.01	0.01	0.01	0.01
Lg.intestine	1.84	0.88	1.1	0.63	0.91	0.04	0.02	0.03	0.04	0.04	0.02	0.02	0.02	0.02	0.02	0.01	0.01	0.01	0.01	0.01
Kidneys	0.98	0.94	0.9	0.86	0.82	0.58	0.5	0.45	0.68	0.79	0.37	0.42	0.51	0.7	0.41	0.4	0.18	0.18	0.23	0.24
Muscle	0.03	0.03	0.02	0.02	0.03	0.01	0.01	0.01	0.02	0.02	0.01	0.01	0.01	0.01	0.01	0.01	0.01	0.01	0.01	0
Tail	0.04	0.04	0.04	0.04	0.03	0.01	0.02	0.01	0.03	0.02	0.01	0	0.02	0.03	0.01	0.01	0.01	0.01	0	0.01
Bone	0.19	0.09	0.13	0.13	0.12	0.3	0.04	0.08	0.05	0.2	0.04	0.11	0.04	0.03	0.05	0.2	0.02	0.02	0.03	0.12
Ovaries	0.1	0.11	0.1	0.07	0.09	0.05	0.03	0.04	0.08	0.03	0.05	0.06	0.05	0.13	0.04	0.08	0.04	0.05	0.03	0.04
Uterus	0.12	0.16	0.16	0.11	0.12	0.04	0.06	0.07	0.12	0.12	0.05	0.15	0.06	0.11	0.1	0.09	0.03	0.19	0.09	0.05

[177Lu]Lu-Gemini, 3.7 MBq, 0.2 nmol (%ID/g) – 24 h interval between antibody and clearing agent injection.

	2 h					24 h					48 h					120 h				
	M1	M2	M3	M4	M5	M1	M2	M3	M4	M5	M1	M2	M3	M4	M5	M1	M2	M3	M4	M5
Blood	0.19	0.55	0.86	0.83	0.9	0.46	0.28	0.06	0.36	0.17	0.02	0.17	0.02	0.02	0.03	0	0	0.01	0.02	0.01
Tumor	7.03	12.22	13.68	9.25	7.74	9.88	8.63	10.19	5.91	11.5	9.64	6.65	10.14	9.79	8.34	3.43	3.75	5.82	7.43	5.93
Heart	0.21	0.38	0.41	0.46	0.49	0.48	0.28	0.17	0.31	0.25	0.15	0.3	0.16	0.22	0.19	0.14	0.16	0.16	0.19	0.17
Lungs	0.64	0.86	1.4	0.89	1.3	1.05	0.48	0.71	0.56	0.49	0.23	0.4	0.26	0.27	0.27	0.15	0.18	0.17	0.23	2.55*
Liver	0.33	0.35	0.46	0.47	0.55	0.73	0.54	0.33	0.68	0.56	0.67	0.72	0.4	0.44	0.41	0.21	0.24	0.35	0.6	0.3
Spleen	0.16	0.34	0.47	0.39	0.47	0.6	0.46	0.22	0.38	0.37	0.37	0.51	0.33	0.31	0.31	0.2	0.23	0.3	0.45	0.31
Stomach	0.06	0.15	0.19	0.22	0.21	0.08	0.09	0.06	0.13	0.08	0.07	0.19	0.08	0.07	0.05	0.04	0.04	0.14	0.08	0.22
Sm.intestine	0.28	0.22	0.31	0.29	0.31	0.15	0.15	0.14	0.14	0.08	0.06	0.16	0.12	0.13	0.09	0.05	0.06	0.17	0.11	0.09
Lg.intestine	0.79	1.27	1.36	0.76	1.04	0.2	0.18	0.56	0.25	0.12	0.08	0.18	0.28	0.37	0.08	0.05	0.1	0.26	0.13	0.23
Kidneys	1.49	1.67	1.92	2.04	1.74	1.34	0.78	0.86	1.17	1.16	0.63	0.93	0.77	0.83	0.89	0.35	0.51	0.53	0.53	0.8
Muscle	0.14	0.12	0.13	0.13	0.14	0.11	0.08	0.18	0.08	0.07	0.09	0.06	0.03	0.05	0.05	0.03	0.03	0.04	0.05	0.04
Tail	0.11	0.17	0.16	0.14	0.23	0.17	0.09	0.09	0.13	0.11	0.07	0.15	0.06	0.08	0.11	0.06	0.04	0.07	0.12	0.06
Bone	0.37	1.19	0.57	0.45	1.09	0.37	0.21	0.34	0.35	0.24	0.17	0.24	0.18	0.31	0.21	0.08	0.08	0.13	0.14	0.11
Ovaries	0.33	0.59	0.93	0.59	0.61	0.87	0.45	0.4	0.6	0.46	0.33	0.5	0.31	0.33	0.24	0.21	0.18	0.33	0.45	0.38
Uterus	0.47	1.57	2.1	1.18	1.91	1.61	0.49	0.56	0.68	0.55	0.38	0.58	0.85	0.58	0.55	0.52	0.38	0.52	0.22	0.44

* The high %ID/g value in the lungs of M5 at 120 h was due to the presence of multiple small pulmonary nodules (most likely due to inadvertent direct hematogenous systemic seeding into the hepatic venous circulation at the time of SW1222-luc inoculation). This value was excluded for the dosimetry estimate.

[177Lu]Lu-Gemini, 3.7 MBq, 0.2 nmol (%ID/g) – 48 h interval between antibody and clearing agent injection.

	2 h					24 h					48 h					120 h				
	M1	M2	M3	M4	M5	M1	M2	M3	M4	M5	M1	M2	M3	M4	M5	M1	M2	M3	M4	M5
Blood	0.31	0.31	0.25	0.34	0.33	0.09	0.05	0.16	0.05	0.08	0.03	0.02	0.03	0.04	0.03	0	0.01	0	0	0
Tumor	13.07	12.22	12.53	16.23	16.03	7.56	10.3	8.89	10.64	10.7	5.29	8.26	9.82	6.73	6.62	5.63	5.22	5.21	4.07	4.3
Heart	0.28	0.29	0.31	0.34	0.41	0.28	0.27	0.25	0.21	0.19	0.18	0.16	0.18	0.22	0.13	0.12	0.2	0.13	0.27	0.13
Lungs	0.68	0.57	0.73	0.74	0.89	1.7	0.5	0.65	0.45	0.29	0.33	0.25	0.45	1.02	0.43	0.15	0.44	0.16	0.98	0.25
Liver	0.25	0.26	0.22	0.31	0.31	0.2	0.25	0.31	0.23	0.27	0.23	0.23	0.28	0.24	0.17	0.16	0.83	0.15	0.21	0.3
Spleen	0.31	0.22	0.44	0.62	0.39	0.26	0.27	0.29	0.2	0.33	0.19	0.24	0.26	0.25	0.23	0.12	0.62	0.18	0.18	0.28
Stomach	0.09	0.06	0.08	0.12	0.16	0.05	0.11	0.06	0.06	0.07	0.04	0.06	0.04	0.08	0.03	0.04	0.02	0.04	0.03	0.03
Sm.intestine	0.16	0.16	0.15	0.35	0.52	0.09	0.08	0.15	0.08	0.1	0.06	0.08	0.09	0.1	0.05	0.04	0.07	0.04	0.04	0.06
Lg.intestine	0.85	0.44	0.4	0.64	0.36	0.08	0.1	0.1	0.09	0.12	0.09	0.12	0.12	0.47	0.05	0.03	0.05	0.03	0.02	0.04
Kidneys	1.55	1.62	1.32	1.42	1.4	1.14	1.08	1.12	1.07	1.11	0.66	0.85	0.71	0.66	0.89	0.45	0.75	0.5	0.51	0.56
Muscle	0.06	0.07	0.07	0.09	0.1	0.04	0.05	0.08	0.06	0.04	0.04	0.03	0.05	0.05	0.03	0.03	0.03	0.03	0.03	0.03
Tail	0.1	0.1	0.06	0.11	0.13	0.06	0.07	0.09	0.07	0.08	0.05	0.03	0.06	0.06	0.04	0.02	0.05	0.02	0.04	0.04
Bone	0.33	0.38	0.35	0.34	0.25	0.15	0.16	0.15	0.24	0.2	0.09	0.08	0.11	0.11	0.07	0.06	0.05	0.08	0.1	0.07
Ovaries	0.32	0.26	0.31	0.32	0.67	0.2	0.25	0.31	0.21	0.21	0.2	0.12	0.29	0.18	0.14	0.16	0.19	0.17	0.14	0.19
Uterus	0.66	0.4	0.49	0.81	0.54	0.35	0.41	0.52	0.25	0.45	0.65	0.3	0.21	0.45	0.52	0.15	0.27	0.31	0.26	0.34

Table S2. Summary of pathologist necropsy findings from 3-step DOTA-PRIT study. All tissues are normal unless described. Significant treatment-related microscopic lesions are shown in red font below.

group	Animal ID	Survival (days)	Anatomic Pathology	
			Gross Findings	Microscopic Findings
**[^{177}Lu]Lu-ABD**	**G1M1**<br><br>**Complete necropsy**	64 days	• Nutritional condition score: 2<br>• Body weight: 28.3 g<br>• Approximately 1.5 ml dark red fluid in abdomen<br>• Spleen mildly enlarged<br>• Diaphragm: 2 masses, 10x6x4 mm, 8x5x3 mm, pale tan<br>• Liver: 2 masses, 13x8x7 mm, 24x20x10 mm, pale tan<br>• Intestines: multiple masses, from 1 mm diameter to 10x6x5 mm, pale tan and red<br>• Multiple enlarged axillary lymph nodes, from 7x4x3 mm to 5x4x3 mm, dark red	• Thymus: thymic cysts<br>• Liver: Large, highly cellular adenocarcinoma composed of viable neoplastic cells, with multifocal necrosis<br>• Gallbladder: metastatic adenocarcinoma<br>• Duodenum, jejunum, ileus: metastatic adenocarcinoma<br>• Salivary glands: lymphocytic infiltrate, multifocal, moderate<br>• Ovaries: atrophy, marked<br>• Skin (trunk): acanthosis and hyperkeratosis, multifocal, mild<br>• Harderian gland: adenitis, neutrophilic and necrotizing, diffuse, unilateral, marked<br>• Axillary lymph node: metastatic adenocarcinoma<br>• Diaphragm: metastatic adenocarcinoma
	**G1M2**<br><br>**Complete necropsy**	64 days	• Nutritional condition score: 2<br>• Body weight: 24.7 g<br>• ~ 0.5 ml dark red fluid in abdomen<br>• Spleen mildly enlarged<br>• Multiple coalescing masses in omentum (near stomach and adhered to liver), 1 to 3 mm in diameter<br>• Liver: 1 mass, 20x16x11 mm<br>• Left axillary lymph node: slightly enlarged (1 mm)	• Lungs: alveolar infiltrate, histiocytic and eosinophilic, focal, mild<br>• Thymus: thymic cysts<br>• Liver: Large, highly cellular adenocarcinoma composed of viable neoplastic cells, with multifocal necrosis<br>• Stomach: serosal fibrosis, focal, moderate<br>• Ovaries: atrophy, marked<br>• Skin (trunk): acanthosis and hyperkeratosis, multifocal, moderate<br>• Harderian gland: adenitis, histiocytic, diffuse, unilateral, moderate
**[^{177}Lu]Lu-Gemini**	**G2M1**<br><br>**Selected organs in formalin**	81 days		• Kidneys: tubular degeneration, focal, unilateral, minimal<br>• Liver: large, highly cellular adenocarcinoma composed of viable neoplastic cells, with multifocal necrosis. Cholangiohepatitis, neutrophilic and histiocytic, with bile duct hyperplasia, portal fibrosis, and random foci of hepatocyte necrosis, multifocal, marked<br>• Gallbladder: metastatic adenocarcinoma<br>• Femur and bone marrow: normal
	**G2M2**<br><br>**Complete necropsy**	113 days	• Nutritional condition score: 2<br>• Body weight: 32.8 g<br>• Large amount of dark red fluid in abdomen<br>• Liver: 1 mass, 20x12x10 mm	• Thymus: thymic cysts<br>• Liver: large, highly cellular adenocarcinoma composed of viable neoplastic cells, with multifocal necrosis<br>• Ovaries: atrophy, marked<br>• Skin (trunk): acanthosis and hyperkeratosis, diffuse, moderate<br>• Harderian gland: adenitis, histiocytic, with fibrosis, multifocal, bilateral, moderate<br>• Ears: otitis externa, neutrophilic, unilateral, mild

	**G2M3**<br><br>**Complete necropsy**	113 days	• Nutritional condition score: 2<br>• Body weight: 26.2 g<br>• Liver: 2 masses, 20x18x18 mm, 4x9x5 mm<br>• Mesentery and pancreas: 1 mass, 11x10x10 mm	• Thymus: thymic cysts<br>• Liver: Large, highly cellular adenocarcinoma composed of viable neoplastic cells, with multifocal necrosis<br>• Stomach: muscularis mineralization, focal,<br>• Pancreas: metastatic adenocarcinoma<br>• Ovaries: atrophy, marked. Ovarian cyst, focal<br>• Skin (trunk): acanthosis and hyperkeratosis, diffuse, moderate<br>• Harderian gland: adenitis, histiocytic, with fibrosis, diffuse, unilateral, moderate
	**G2M1**<br><br>**Selected organs in formalin**	81 days		• Kidneys: focally extensive cortical degeneration, atrophy, and fibrosis (consistent with renal infarct), unilateral<br>• Liver: focal scar composed of fibrosis, necrotic debris, mucin, and granulomatous inflammation. No evidence of viable tumor cells. Hepatocytes: glycogen depletion and minimal lipidosis, diffuse<br>• Femur and bone marrow: normal
	**G2M5**<br><br>**Complete necropsy**	68 days	• Nutritional condition score: 2<br>• Body weight: 20.5 g<br>• Liver: 1 mass, 3 mm in diameter<br>• Epaxial musculature appears swollen in lumbar region of spine	• Kidneys: glomerulonephritis, membranous, multifocal, bilateral, moderate. Tubular degeneration, multifocal, bilateral, mild<br>• Liver: small, poorly cellular adenocarcinoma, composed of mostly of fibrosis, necrotic debris, mucin, and granulomatous inflammation, with small numbers of viable neoplastic cells<br>• Uterus mesometrium: metastatic adenocarcinoma, composed of mostly of fibrosis and calcification, with small numbers of viable neoplastic cells<br>• Ovaries: atrophy, marked<br>• Skin (trunk): acanthosis and hyperkeratosis, diffuse, mild<br>• Spinal cord: metastatic adenocarcinoma<br>• Harderian gland: adenitis, histiocytic, multifocal, bilateral, mild
**Control group**	**G3M1**<br><br>**Selected organs in formalin**	18 days		• Kidneys: normal<br>• Liver: large, highly cellular adenocarcinoma composed of viable neoplastic cells, with multifocal necrosis<br>• Femur and bone marrow: normal
	**G3M2**<br><br>**Complete necropsy**	18 days	• Nutritional condition score: 2<br>• Body weight: not recorded<br>• Liver, diffusely pale, containing one mass, 19 x 13 x 12 mm, mottled dark red and tan.<br>• Scaly skin on dorsal trunk and neck regions<br>• Eyes: one eye is not observed and is replaced by abscess (see Harderian gland)	• Heart right ventricular lumen: adenocarcinoma embolus<br>• Lungs: metastatic adenocarcinoma, multifocal<br>• Thymus: thymic cysts<br>• Liver: large, highly cellular adenocarcinoma composed of viable neoplastic cells, with multifocal necrosis. Hepatocytes: glycogen depletion and mild lipidosis, diffuse<br>• Uterus mesometrium: metastatic adenocarcinoma<br>• Skin (trunk): acanthosis and hyperkeratosis, diffuse, moderate

				• Harderian gland: adenitis, neutrophilic and necrotizing, with orbital abscess, diffuse, unilateral, marked
	**G3M3**<br><br>**Complete necropsy**	11 days	• Nutritional condition score: 1<br>• Body weight: 22.2 g<br>• Liver, diffusely pale, containing multiple coalescing masses from 4 to 12 mm in diameter<br>• Abdominal wall: one mass 4 mm in diameter	• Thymus: thymic cysts<br>• Kidneys: tubular basophilia, focal, unilateral, minimal<br>• Liver: large, highly cellular adenocarcinoma composed of viable neoplastic cells, with multifocal necrosis. Hepatocytes: glycogen depletion and mild lipidosis, diffuse.<br>• Skin (trunk): acanthosis and hyperkeratosis, diffuse, moderate<br>• Harderian gland: adenitis, histiocytic, focal, unilateral, mild.<br>• Abdominal wall: Large, highly cellular adenocarcinoma composed of viable neoplastic cells, with multifocal necrosis
	**G3M4**<br><br>**Selected organs in formalin**	11 days		• Kidneys: normal<br>• Liver: Large, highly cellular adenocarcinoma composed of viable neoplastic cells, with multifocal necrosis. Hepatic necrosis, multifocal, random, mild<br>• Femur and bone marrow: normal
	**G3M5**<br><br>**Selected organs in formalin**	18 days		• Kidneys: normal<br>• Liver: Large, highly cellular adenocarcinoma composed of viable neoplastic cells, with multifocal necrosis. Hepatic necrosis, multifocal, random, mild<br>• Femur and bone marrow: normal

REFERENCES

1. Cheal SM, Xu H, Guo HF, Zanzonico PB, Larson SM, Cheung NK. Preclinical evaluation of multistep targeting of diasialoganglioside GD2 using an IgG-scFv bispecific antibody with high affinity for GD2 and DOTA metal complex. Mol Cancer Ther. 2014; 13: 1803-12.
2. Dacek MM, Veach DR, Cheal SM, Carter LM, McDevitt MR, Punzalan B, et al. Engineered Cells as a Test Platform for Radiohaptens in Pretargeted Imaging and Radioimmunotherapy Applications. Bioconjug Chem. 2021; 32: 649-54.
3. Orcutt KD, Nasr KA, Whitehead DG, Frangioni JV, Wittrup KD. Biodistribution and clearance of small molecule hapten chelates for pretargeted radioimmunotherapy. Mol Imaging Biol. 2011; 13: 215-21.
4. Vaughn BA, Lee SG, Vargas DB, Seo S, Rinne SS, Xu H, et al. Theranostic GPA33-Pretargeted Radioimmunotherapy of Human Colorectal Carcinoma with a Bivalent (177)Lu-Labeled Radiohapten. J Nucl Med. 2024.
5. Payne KM, Woods M. Isomerism in benzyl-DOTA derived bifunctional chelators: implications for molecular imaging. Bioconjug Chem. 2015; 26: 338-44.
6. Mannheim JG, Schlichthaerle T, Kuebler L, Quintanilla-Martinez L, Kohlhofer U, Kneilling M, et al. Comparison of small animal CT contrast agents. Contrast Media Mol Imaging. 2016; 11: 272-84.
7. Rader C, Ritter G, Nathan S, Elia M, Gout I, Jungbluth AA, et al. The rabbit antibody repertoire as a novel source for the generation of therapeutic human antibodies. J Biol Chem. 2000; 275: 13668-76.
